# A *C6orf10*/*LOC101929163* locus is associated with age of onset in *C9orf72* carriers

**DOI:** 10.1093/brain/awy238

**Published:** 2018-09-25

**Authors:** Ming Zhang, Raffaele Ferrari, Maria Carmela Tartaglia, Julia Keith, Ezequiel I Surace, Uri Wolf, Christine Sato, Mark Grinberg, Yan Liang, Zhengrui Xi, Kyle Dupont, Philip McGoldrick, Anna Weichert, Paul M McKeever, Raphael Schneider, Michael D McCorkindale, Claudia Manzoni, Rosa Rademakers, Neill R Graff-Radford, Dennis W Dickson, Joseph E Parisi, Bradley F Boeve, Ronald C Petersen, Bruce L Miller, William W Seeley, John C van Swieten, Jeroen van Rooij, Yolande Pijnenburg, Julie van der Zee, Christine Van Broeckhoven, Isabelle Le Ber, Vivianna Van Deerlin, EunRan Suh, Jonathan D Rohrer, Simon Mead, Caroline Graff, Linn Öijerstedt, Stuart Pickering-Brown, Sara Rollinson, Giacomina Rossi, Fabrizio Tagliavini, William S Brooks, Carol Dobson-Stone, Glenda M Halliday, John R Hodges, Olivier Piguet, Giuliano Binetti, Luisa Benussi, Roberta Ghidoni, Benedetta Nacmias, Sandro Sorbi, Amalia C Bruni, Daniela Galimberti, Elio Scarpini, Innocenzo Rainero, Elisa Rubino, Jordi Clarimon, Alberto Lleó, Agustin Ruiz, Isabel Hernández, Pau Pastor, Monica Diez-Fairen, Barbara Borroni, Florence Pasquier, Vincent Deramecourt, Thibaud Lebouvier, Robert Perneczky, Janine Diehl-Schmid, Jordan Grafman, Edward D Huey, Richard Mayeux, Michael A Nalls, Dena Hernandez, Andrew Singleton, Parastoo Momeni, Zhen Zeng, John Hardy, Janice Robertson, Lorne Zinman, Ekaterina Rogaeva, International FTD-Genomics Consortium (IFGC), Raffaele Ferrari, Dena G Hernandez, Michael A Nalls, Jonathan D Rohrer, Adaikalavan Ramasamy, John B J Kwok, Carol Dobson-Stone, William S Brooks, Peter R Schofield, Glenda M Halliday, John R Hodges, Olivier Piguet, Lauren Bartley, Elizabeth Thompson, Isabel Hernández, Agustín Ruiz, Mercè Boada, Barbara Borroni, Alessandro Padovani, Carlos Cruchaga, Nigel J Cairns, Luisa Benussi, Giuliano Binetti, Roberta Ghidoni, Gianluigi Forloni, Diego Albani, Daniela Galimberti, Chiara Fenoglio, Maria Serpente, Elio Scarpini, Jordi Clarimón, Alberto Lleó, Rafael Blesa, Maria Landqvist Wald&ouml, Karin Nilsson, Christer Nilsson, Ian R A Mackenzie, Ging-Yuek R Hsiung, David M A Mann, Jordan Grafman, Christopher M Morris, Johannes Attems, Timothy D Griffiths, Ian G McKeith, Alan J Thomas, Pietro Pietrini, Edward D Huey, Eric M Wassermann, Atik Baborie, Evelyn Jaros, Michael C Tierney, Pau Pastor, Cristina Razquin, Sara Ortega-Cubero, Elena Alonso, Robert Perneczky, Janine Diehl-Schmid, Panagiotis Alexopoulos, Alexander Kurz, Innocenzo Rainero, Elisa Rubino, Lorenzo Pinessi, Ekaterina Rogaeva, Peter St George-Hyslop, Giacomina Rossi, Fabrizio Tagliavini, Giorgio Giaccone, James B Rowe, Johannes C M Schlachetzki, James Uphill, John Collinge, Simon Mead, Adrian Danek, Vivianna M Van Deerlin, Murray Grossman, John Q Trojanowski, Julie van der Zee, Christine Van Broeckhoven, Stefano F Cappa, Isabelle Leber, Didier Hannequin, Véronique Golfier, Martine Vercelletto, Alexis Brice, Benedetta Nacmias, Sandro Sorbi, Silvia Bagnoli, Irene Piaceri, Jørgen E Nielsen, Lena E Hjermind, Matthias Riemenschneider, Manuel Mayhaus, Bernd Ibach, Gilles Gasparoni, Sabrina Pichler, Wei Gu, Martin N Rossor, Nick C Fox, Jason D Warren, Maria Grazia Spillantini, Huw R Morris, Patrizia Rizzu, Peter Heutink, Julie S Snowden, Sara Rollinson, Anna Richardson, Alexander Gerhard, Amalia C Bruni, Raffaele Maletta, Francesca Frangipane, Chiara Cupidi, Livia Bernardi, Maria Anfossi, Maura Gallo, Maria Elena Conidi, Nicoletta Smirne, Rosa Rademakers, Matt Baker, Dennis W Dickson, Neill R Graff-Radford, Ronald C Petersen, David Knopman, Keith A Josephs, Bradley F Boeve, Joseph E Parisi, William W Seeley, Bruce L Miller, Anna M Karydas, Howard Rosen, John C van Swieten, Elise G P Dopper, Harro Seelaar, Yolande A L Pijnenburg, Philip Scheltens, Giancarlo Logroscino, Rosa Capozzo, Valeria Novelli, Annibale A Puca, Massimo Franceschi, Alfredo Postiglione, Graziella Milan, Paolo Sorrentino, Mark Kristiansen, Huei-Hsin Chiang, Caroline Graff, Florence Pasquier, Adeline Rollin, Vincent Deramecourt, Thibaud Lebouvier, Dimitrios Kapogiannis, Luigi Ferrucci, Stuart Pickering-Brown, Andrew B Singleton, John Hardy, Parastoo Momeni

**Affiliations:** 1 Shanghai First Rehabilitation Hospital, School of Medicine, Tongji University, Shanghai, China; 2 Institute for Advanced Study, Tongji University, Shanghai, China; 3 Tanz Centre for Research in Neurodegenerative Diseases, University of Toronto, Toronto, ON, Canada; 4 Department of Molecular Neuroscience, Institute of Neurology, UCL, London, UK; 5 Krembil Neuroscience Center, University Health Network Memory clinic, Toronto Western Hospital, Toronto, ON, Canada; 6 Department of Medicine, Division of Neurology, University of Toronto, Toronto, ON, Canada; 7 Sunnybrook Health Sciences Centre, University of Toronto, Toronto, ON, Canada; 8 Laboratorio de Biología Molecular, Departamento de Neuropatología, Instituto de Investigaciones Neurológicas Dr. Raúl Carrea (FLENI), Buenos Aires, Argentina; 9 Baycrest Health Science, Department of Psychiatry, University of Toronto, Toronto, ON, Canada; 10 School of Pharmacy, University of Reading, Whiteknights, Reading, UK; 11 Department of Neuroscience, Mayo Clinic, Jacksonville, FL, USA; 12 Department of Neurology, Mayo Clinic, Jacksonville, FL, USA; 13 Department of Laboratory Medicine and Pathology and Department of Neurology, Mayo Clinic, Rochester, MN, USA; 14 Department of Neurology, Mayo Clinic, Rochester, MN, USA; 15 Department of Neurology, University of California San Francisco Memory and Aging Center, San Francisco, CA, USA; 16 Department of Neurology and Department of Pathology, University of California San Francisco Memory and Aging Center, San Francisco, CA, USA; 17 Department of Neurology, Erasmus MC, Rotterdam, The Netherlands; 18 Alzheimer Center, VU University Medical Center, Amsterdam Neuroscience, Amsterdam, The Netherlands; 19 Neurodegenerative Brain Diseases, Center of Molecular Neurology, VIB, Antwerp, Belgium; 20 Laboratory of Neurogenetics, Institute Born-Bunge, University of Antwerp, Antwerp, Belgium; 21 Sorbonne Universités, UPMC Univ Paris 06, Inserm U1127, CNRS UMR 7225, Institut du Cerveau et la Moelle épinière (ICM), Paris, France; 22 Reference Center for Rare and Young Dementias, Institute of Memory and Alzheimer’s Disease (IM2A), Department of Neurology, Hopital Pitié-Salpêtrière, Paris, France; 23 Center for Neurodegenerative Disease Research, Department of Pathology and Laboratory Medicine, Perelman School of Medicine at the University of Pennsylvania, Philadelphia, PA, USA; 24 Dementia Research Centre, Department of Neurodegenerative Disease, UCL Institute of Neurology, London, UK; 25 MRC Prion Unit at UCL, Institute of Prion Diseases, London, UK; 26 Division of Neurogeriatrics, Alzheimer Research Center, Karolinska Institutet, Solna, Sweden; 27 Genetics Unit, Theme Aging, Karolinska University Hospital, Stockholm, Sweden; 28 Division of Neuroscience and Experimental Psychology, School of Biological Sciences, Faculty of Biology, Medicine and Health, University of Manchester, University of Manchester, UK; 29 Division of Neurology V and Neuropathology, Fondazione IRCCS Istituto Neurologico Carlo Besta, Milano, Italy; 30 Scientific Directorate, Fondazione IRCCS Istituto Neurologico Carlo Besta, Milano, Italy; 31 Neuroscience Research Australia and Prince of Wales Clinical School, University of New South Wales, Sydney, Australia; 32 Brain and Mind Centre, Sydney Medical School, The University of Sydney, Sydney, Australia; 33 School of Medical Sciences, University of New South Wales, Sydney, Australia; 34 Australian Research Council Centre of Excellence in Cognition and its Disorders, Sydney, Australia; 35 School of Psychology and Brain and Mind Centre, University of Sydney, Sydney, Australia; 36 MAC Memory Center, IRCCS Istituto Centro San Giovanni di Dio Fatebenefratelli, Brescia, Italy; 37 Molecular Markers Laboratory, IRCCS Istituto Centro San Giovanni di Dio Fatebenefratelli, Brescia, Italy; 38 Department of Neuroscience, Psychology, Drug Research and Child Health, University of Florence, Florence, Italy; 39 IRCCS Don Gnocchi, Florence, Italy; 40 Regional Neurogenetic Centre, Lamezia Terme, Azienda Sanitaria Provinciale Catanzaro, Italy; 41 Neurodegenerative Disease Unit, University of Milan, Fondazione Ca’ Granda, IRCCS Ospedale Policlinico, Milan, Italy; 42 Department of Neuroscience “Rita Levi Montalcini”, University of Torino, Torino, Italy; 43 IIB-Sant Pau, Hospital de la Santa Creu i Sant Pau, Universitat Autonoma de Barcelona, Barcelona, Spain; 44 Centre of Biomedical Investigation Network for Neurodegenerative Diseases (CIBERNED), Madrid, Spain; 45 Research Center and Memory Clinic, Fundació ACE, Institut Català de Neurociències Aplicades-Universitat Internacional de Catalunya, Barcelona, Spain; 46 Memory Disorders Unit, Department of Neurology, Hospital Universitari Mutua de Terrassa, Barcelona, Spain; 47 Fundació per la Recerca Biomèdica i Social Mútua de Terrassa, Terrassa, Barcelona, Spain; 48 Centre for Neurodegenerative Disorders, Department of Clinical and Experimental Sciences, University of Brescia, Brescia, Italy; 49 National Reference Center for Young Onset Dementia, Neurology Department, Centre Hospitalier Régional Universitaire de Lille, University Hospital, Inserm U1171, DistAlz, Lille, France; 50 Department of Psychiatry and Psychotherapy, Technische Universität München, Munich, Germany; 51 Department of Psychiatry and Psychotherapy, Division of Mental Health in Older Adults and Alzheimer Therapy and Research Center, Ludwig-Maximilians-Universität München, Munich, Germany; 52 Imperial College London, School of Public Health, Neuroepidemiology and Ageing Research Unit, London, UK; 53 Cognitive Neurology and Alzheimer’s Center, Department of Psychiatry, Feinberg School of Medicine Chicago, IL, USA; 54 Department of Psychology, Weinberg College of Arts and Sciences Northwestern University Chicago, IL, USA; 55 The Taub Institute for Research on Alzheimer’s Disease and the Aging Brain, Columbia University Medical Center, New York, NY, USA; 56 The Gertrude H. Sergievsky Center, The Departments of Neurology, Psychiatry, Epidemiology, School of Public Health, Columbia University, New York, NY, USA; 57 Laboratory of Neurogenetics, National Institute on Aging, Bethesda, MD, USA; 58 Rona Holdings, Silicon Valley, CA, USA; 59 Merck & Co., Inc, Kenilworth, NJ, USA

**Keywords:** *C9orf72*, genetic association, age of onset, amyotrophic lateral sclerosis, frontotemporal dementia

## Abstract

The G_4_C_2_-repeat expansion in *C9orf72* is the most common known cause of amyotrophic lateral sclerosis and frontotemporal dementia. The high phenotypic heterogeneity of *C9orf72* patients includes a wide range in age of onset, modifiers of which are largely unknown. Age of onset could be influenced by environmental and genetic factors both of which may trigger DNA methylation changes at CpG sites. We tested the hypothesis that age of onset in *C9orf72* patients is associated with some common single nucleotide polymorphisms causing a gain or loss of CpG sites and thus resulting in DNA methylation alterations. Combined analyses of epigenetic and genetic data have the advantage of detecting functional variants with reduced likelihood of false negative results due to excessive correction for multiple testing in genome-wide association studies. First, we estimated the association between age of onset in *C9orf72* patients (*n = *46) and the DNA methylation levels at all 7603 CpG sites available on the 450 k BeadChip that are mapped to common single nucleotide polymorphisms. This was followed by a genetic association study of the discovery (*n = *144) and replication (*n = *187) *C9orf72* cohorts. We found that age of onset was reproducibly associated with polymorphisms within a 124.7 kb linkage disequilibrium block tagged by top-significant variation, rs9357140, and containing two overlapping genes (*LOC101929163* and *C6orf10*). A meta-analysis of all 331 *C9orf72* carriers revealed that every A-allele of rs9357140 reduced hazard by 30% (*P = *0.0002); and the median age of onset in AA-carriers was 6 years later than GG-carriers. In addition, we investigated a cohort of *C9orf72* negative patients (*n = *2634) affected by frontotemporal dementia and/or amyotrophic lateral sclerosis; and also found that the AA-genotype of rs9357140 was associated with a later age of onset (adjusted *P = *0.007 for recessive model). Phenotype analyses detected significant association only in the largest subgroup of patients with frontotemporal dementia (*n = *2142, adjusted *P = *0.01 for recessive model). Gene expression studies of frontal cortex tissues from 25 autopsy cases affected by amyotrophic lateral sclerosis revealed that the G-allele of rs9357140 is associated with increased brain expression of *LOC101929163* (a non-coding RNA) and *HLA-DRB1* (involved in initiating immune responses), while the A-allele is associated with their reduced expression. Our findings suggest that carriers of the rs9357140 GG-genotype (linked to an earlier age of onset) might be more prone to be in a pro-inflammatory state (e.g. by microglia) than AA-carriers. Further, investigating the functional links within the *C6orf10*/*LOC101929163*/*HLA-DRB1* pathway will be critical to better define age-dependent pathogenesis of frontotemporal dementia and amyotrophic lateral sclerosis.

## Teaser

Discovery of disease age-of-onset modifiers is important for clinical trials and drug design. Zhang *et al*. perform a genome-wide analysis of epigenetic functional polymorphisms and identify an association between the *C6orf10/LOC101929163* locus and age of FTD/ALS onset. The risk allele may be associated with a pro-inflammatory state in the brain.

## Introduction

The G_4_C_2_-repeat expansion in *C9orf72* is the most common known cause of amyotrophic lateral sclerosis (ALS) and frontotemporal dementia (FTD) (DeJesus-Hernandez *et al.*, 2011; Renton *et al.*, 2011; Gijselinck *et al.*, 2012) in Caucasians. It accounts for about 37% familial and 7% sporadic ALS patients; as well as 25% familial and 6% sporadic FTD patients (Rademakers, 2012) with age and sex dependent disease penetrance (Murphy *et al.*, 2017). High phenotypic heterogeneity of *C9orf72* patients also includes a wide range in disease age of onset (27–74 years) and duration (0.5–22 years) (Gijselinck *et al.*, 2016). Yet, genetic modifiers of age of onset in *C9orf72* patients are largely unknown [only the T-allele of rs1990622 in *TMEM106B* was associated with a later age of onset of FTD, but not ALS (Gallagher *et al.*, 2014; van Blitterswijk *et al.*, 2014)]. Detection of the age of onset modifier(s) might increase the accuracy of predicting age of onset in asymptomatic mutation carriers, which is important for clinical trials focused on early intervention.

Age of onset could be influenced by genetic and environmental modifiers, both of which may trigger epigenetic changes, such as DNA methylation at CpG sites (Zhang *et al.*, 2016). Indeed, there is no a strict dichotomy between action of genetic and epigenetic factors; they often work in concert. Genome-wide DNA methylation profiles of identical twins are much more similar than between fraternal siblings (Zhang *et al.*, 2016), demonstrating that many epigenetic changes are genetically controlled (e.g. the repeat expansion causes hypermethylation of the *C9orf72* locus leading to downregulation of *C9orf72* expression) (Xi *et al.*, 2015*b*; Gijselinck *et al.*, 2016). The DNA methylation levels of some CpGs are age-related allowing the estimation of DNA methylation age based on the cumulative assessment of 353 CpGs included on the genome-wide 450K BeadChip. Currently, DNA methylation age is the most accurate predictor of chronological age across multiple tissues (Horvath, 2013), but may in fact reflect biological age better than chronological age. Indeed, we recently reported that increased DNA methylation age acceleration (DNA methylation age minus chronological age) is associated with earlier age of onset in *C9orf72* patients analysed on the 450 K BeadChip after exclusion of CpGs mapped to common single nucleotide polymorphisms (SNPs) (Zhang *et al.*, 2017).

CpGs are the most mutable sites in the human genome because methyl-C can spontaneously deaminate to T (e.g. 35% of all coding mutations occur at CpG sites) (Lek *et al.*, 2016). Hence, in the current study we tested the hypothesis that age of onset in *C9orf72* patients is associated with some common SNPs causing a gain or loss of CpG sites (CpG-SNPs) and thus resulting in DNA methylation changes. Allele-specific DNA methylation is largely attributed to CpG-SNPs (Shoemaker *et al.*, 2010), which have often been detected within promoter regions, transcription factor binding sites and DNase I hypersensitive sites (Gagliano *et al.*, 2016), thus regulating the level of gene expression. CpG-SNPs belong to a group of methylation quantitative trait loci (Hannon *et al.*, 2016), which are linked to some mental disorders (Gagliano *et al.*, 2016).

We combined epigenetic and genetic approaches to map functional variants (CpG-SNPs) associated with age of onset in *C9orf72* carriers. Such a study design reduces the likelihood of false negative results due to excessive correction for multiple testing in genome-wide association studies (GWASs). Our study also includes suggestions on how the significant SNPs exert their effects (e.g. by influencing gene expression).

## Materials and methods

### Participants

Informed consent was obtained from all participants in accordance with the respective ethics review boards. Sample characteristics are presented in Table 1 and Supplementary Table 1 for *C9orf72* carriers, and Supplementary Table 2 for *C9orf72* negative patients. Briefly, our study included patients diagnosed with bulbar or limb onset ALS, behavioural FTD (bvFTD), semantic dementia, progressive non-fluent aphasia (PNFA), and FTD-ALS. All patients were of Caucasian origin and diagnosed at hospitals specializing in neurodegenerative disorders using established clinical criteria for ALS (Brooks *et al.*, 2000) and FTD (Neary *et al.*, 1998), including the revised diagnostic criteria for bvFTD (Rascovsky *et al.*, 2011) and language variants of FTD (Gorno-Tempini *et al.*, 2011). Age of onset was defined as the age at which the first disease symptoms appeared, including initial bulbar or limb symptoms in ALS, and cognitive dysfunction in judgement, language, memory, or changes in behaviour or personality in FTD. Age of onset was either self-reported (for ALS) or obtained from unaffected family members (for FTD).

**Table 1:** Sample characteristics of the discovery and replication *C9orf72* datasets

	Discovery cohort			Replication cohort
	Unrelated carriers	Symptomatic carriers from 16 families	Asymptomatic carriers from 16 families	Unrelated carriers
Number of cases	101	21	22	187
Sex, male, *n* (%)	55 (54.4)	10 (47.6)	12 (45.5)	104 (55.6)
Age of onset, years, median (IQR)	59 (54–66)	55 (48–60)	NA	58 (51–63)
Age of onset, years, mean (range)	59.82 (37–78)	54.86 (38–73)	NA	57.2 (34–80)

NA = not applicable.

The discovery cohort was recruited from Canada, Italy, Spain, UK, USA or Argentina and consisted of 144 *C9orf72* carriers, including 21 symptomatic and 22 asymptomatic carriers from 16 pedigrees. The independent replication cohort was obtained from centres (different from those that collected the discovery cohort) participating in the International FTD-Genomics Consortium (IFGC; https://ifgcsite.wordpress.com/) (Ferrari *et al.*, 2014). It consisted of 187 unrelated FTD or FTD-ALS *C9orf72* carriers from the USA, Canada, UK, France, Belgium, Italy, Germany, Spain, Sweden, the Netherlands and Australia. Information about family relatedness was obtained from the clinical notes of the neurologists who collected the samples. In addition, the presence of relatedness in the replication cohort was previously assessed as part of a GWAS that identified and excluded all first-degree relatives (through identity by descent for any pair with an estimate <0.125) (Ferrari *et al.*, 2014).

For a follow-up study of unrelated *C9orf72* negative patients, we investigated 2142 FTD and 164 FTD-ALS patients from the IFGC (Ferrari *et al.*, 2014), as well as 328 sporadic ALS patients from the ALS clinic at Sunnybrook Health Sciences Centre, Toronto (Supplementary Table 2), which also provided frontal cortex from 25 unrelated ALS autopsy cases without an expansion in *C9orf72* (<30 repeats) for the gene expression studies (Supplementary Table 3).

### Procedures

Blood genomic DNA was extracted using a QIAGEN kit. First, we analysed the genome-wide DNA methylation data from the 450K BeadChip (Illumina) that was previously generated using bisulfite converted DNA of 46 Canadian *C9orf72* carriers (Zhang *et al.*, 2017) to discover common CpG-SNPs with minor allele frequencies >5% that are associated with age of onset. The raw data were preprocessed and analysed using the minfi package in R-project (Aryee *et al.*, 2014). The β-value was used to estimate the DNA methylation level of each CpG-site (β-value of 0: non-methylated; β-value of 1: completely methylated).

All participants of the discovery and replication cohorts (*n = *331) were carriers of an expansion in *C9orf72* (>30 repeats) based on previous analysis by repeat-primed PCR (Ferrari *et al.*, 2014; Xi *et al.*, 2015*b*). Genotypes for rs9357140, rs2143466 and rs1990622 were obtained by Sanger sequencing in the discovery cohort (Supplementary Table 4). For the replication dataset, these SNPs together with eight SNPs in strong linkage disequilibrium (LD) with rs9357140 and rs2143466 (R^2 ^> 0.9) were either genotyped or imputed using the latest data from the Haplotype Reference Consortium (McCarthy *et al.*, 2016) (Supplementary Table 5).

Genotypes for rs9357140 in a follow-up cohort of 2634 unrelated *C9orf72* negative patients with ALS, FTD or FTD-ALS, were obtained by TaqMan^TM^ assay (C___9782529_10, ThermoFisher Scientific) for 328 ALS patients, or imputed from IFGC-GWAS for 2306 FTD and FTD-ALS patients (Ferrari *et al.*, 2014) using the latest data from the Haplotype Reference Consortium (McCarthy *et al.*, 2016).

To measure the degree of LD, we extracted R^2^ values (range from 0 to 1 with higher values indicating a higher LD) from the LDlink tool (https://analysistools.nci.nih.gov/LDlink) using the 1000 Genomes European population data. We searched for known variants within the boundaries of the LD block (R^2 ^> 0.8) tagged by the top significant SNP (rs9357140) using the ‘proxy search’ in LDlink. Functional predictions for the missense SNPs were based on the PolyPhen-2 and SIFT data available from the Exome Aggregation Consortium database (Lek *et al.*, 2016). Using the UCSC genome browser, the LD-block was also analysed for transcriptional factor binding sites and DNase I hypersensitivity.

To detect genes whose expression is associated with rs9357140, we searched for expression quantitative trait loci (eQTL) using Genotype-Tissue Expression (GTEx v7) data from 48 types of human tissues (GTEx Consortium *et al.*, 2017). The GTEx portal (https://www.gtexportal.org/) was used to analyse the association between rs9357140 genotypes and gene expression by a linear regression method. Normalized effect size (NES) was defined as the slope of the linear regression.

To quantify gene expression, total RNA was extracted from human frontal cortex of ALS cases without *C9orf72* expansions using the QIAzol plus RNeasy® Mini Kit (QIAGEN) and reverse transcribed to cDNA using oligo dT primers and the AffinityScript Multiple Temperature cDNA Synthesis Kit (Agilent Technologies). Quantitative RT-PCR was conducted for 25 samples (Supplementary Table 3) with an RNA integrity number >6.5 (based on an Agilent 2100 Bioanalyzer). To select endogenous control genes for the frontal cortex, we assessed four housekeeping genes including *HPRT1* (MIM: 308000; Hs99999909_m1), *UBC* (MIM: 191340; Hs00824723_m1), *B2M* (MIM: 109700; Hs99999907_m1), and *RPLP0* (MIM: 180510; Hs00420895_gH) (ThermoFisher Scientific) in nine samples (*n = *3 per each rs9357140 genotype). We used Normfinder (Andersen *et al.*, 2004) to identify the least variable housekeeping genes (*B2M* and *RPLP0*) in our samples (Supplementary Table 6). We measured expression of *HLA-DRB1* transcript variant 1 (MIM:142857; Hs04192464_mH) and all *C9orf72* transcripts (MIM:614260; Hs00376619_m1) (ThermoFisher Scientific) in triplicate for 25 samples with different rs9357140 genotypes: AA (*n = *9), AG (*n = *8) and GG (*n = *8). Relative quantification was calculated with the ddCt method by geometric mean of housekeeping gene expression (*B2M* and *RPLP0*).

### Statistical analyses

We used the linear regression model of the R minfi package to assess the genome-wide association between the DNA methylation status of CpG-SNPs and age of onset in *C9orf72* patients, as well as to evaluate the false discovery rate to generate adjusted q-values (Zhang *et al.*, 2017). We used a Manhattan plot to prioritize significant variants (*P < *0.01 and q < 0.05) for further genetic study, and a Q-Q plot to highlight potential confounders using the R qqman package (Turner, 2018).

To assess if genotypes affect age of onset, we used a Cox proportional hazard regression model (R survival and survminer packages) (Grambsch, 2000) adjusting for sex, rs1990622 genotypes, disease phenotypes, and censoring age of last follow-up for the 22 currently asymptomatic *C9orf72* carriers. To adjust for relatedness in the Cox proportional hazard regression analysis of the discovery cohort, we created an indicator number for each family; then used the coxph function of the R coxme package with a frailty approach (Ripatti and Palmgren, 2000). The hazard ratio (HR) with 95% confidence interval (CI) is presented. To analyse the association between genotypes and age of onset in the *C9orf72* disease subgroups, we used multivariate linear regression with an additive, dominant or recessive model adjusting for sex, rs1990622 genotypes, or DNA methylation age-acceleration. We also used multivariate linear regression to analyse the association between genotypes and age of onset in *C9orf72* negative disease subgroups (adjusting for sex). We present the linear regression coefficient (B) with standard error (SE) and percentage of response variance explained by the linear regression model (r^2^). Results of additive model were presented, unless otherwise specified.

We used a meta-analysis (R metafor package) with a fixed-effect model to assess the pooled effect size of the Cox regression coefficient (logHR) from the discovery and replication stages (Trinh *et al.*, 2016). We performed a trend analysis using the Cochran–Armitage test to analyse if rs9357140 genotypes are associated with *C9orf72* disease subgroups. A non-parametric Mann-Whitney U-test or Kruskal-Wallis test was used to assess differences in age of onset or gene expression among two or more groups where appropriate. Sex and rs1990622 genotype adjusted *P*-values are shown, unless otherwise specified. The results with *P < *0.05 were accepted as statistically significant.

### Data availability

The data that support the findings of this study are available on request from the corresponding authors (E.R., M.Z.). The data are not publicly available because of information that could compromise the privacy of the research participants.

## Results

### Epigenetic analysis suggested CpG-SNPs associated with age of onset

**Figure 1:**
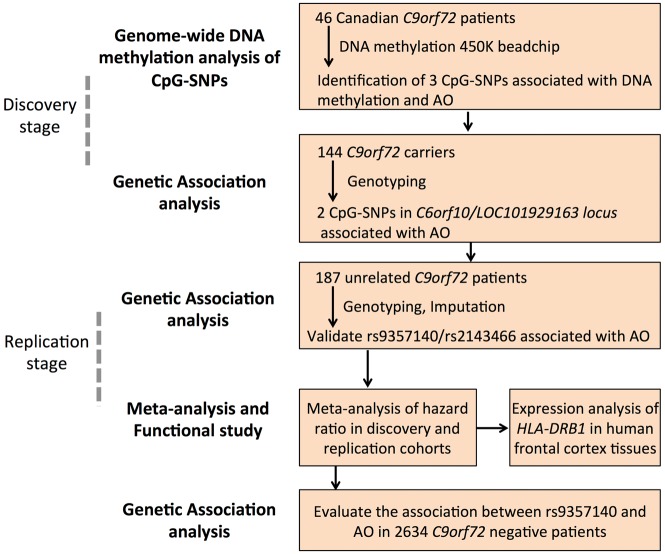
**Flow chart of the study design.** AO = age of onset.

**Figure 2:**
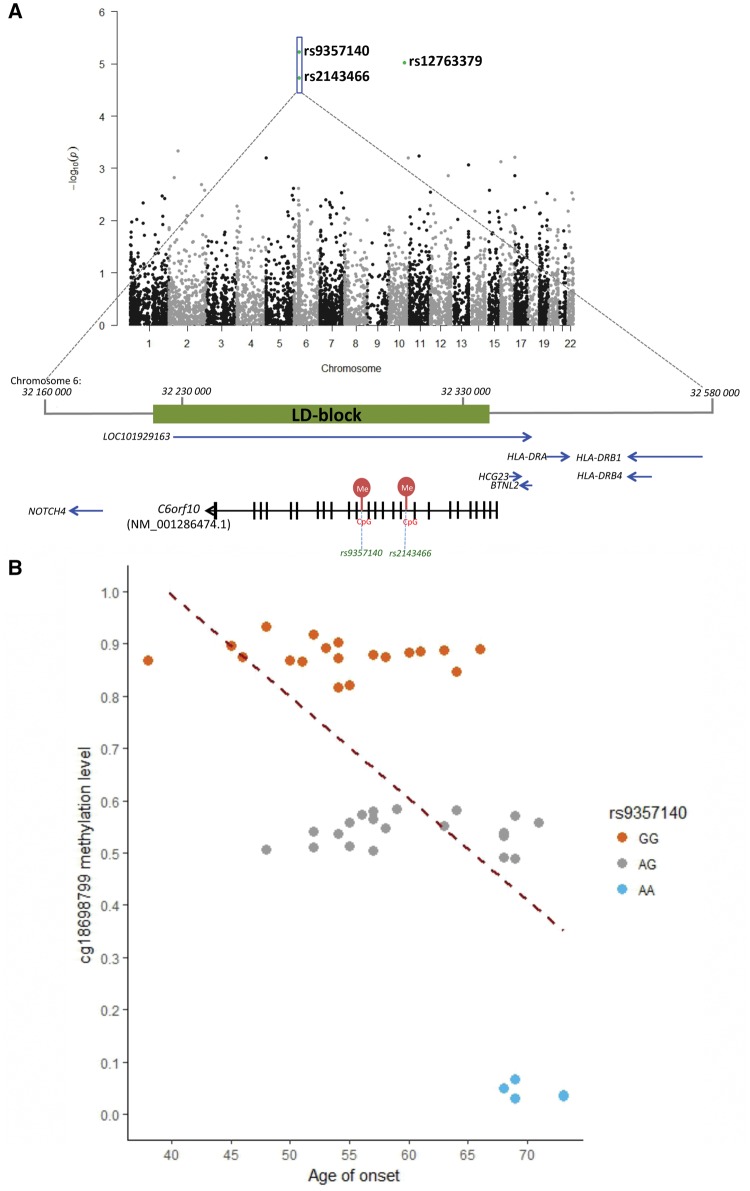
**Genome-wide DNA methylation analysis of the CpG-SNPs in *C9orf72* patients.** (**A**) Manhattan plot presenting the association between DNA methylation status of CpG-SNPs and age of onset, including a locus on chr6:32160000–32580000 with two age of onset-associated CpG-SNPs (rs9357140 and rs2143466 indicated by the box). Arrows indicate the transcriptional direction of each gene (5′ to 3′). ‘Me’ in red represent methylation sites controlled by rs9357140 and rs2143466. The LD block tagged by rs9357140 (R^2 ^> 0.8) is highlighted in green. (**B**) Genotypes of rs9357140 are significantly associated with DNA methylation status: *P* < 1.0 × 10^−6^, B = −0.39 (SE: 0.01); and age of onset: *P* = 2.2 × 10^−5^ adjusted for sex and rs1990622 genotypes, B = 7.01 (SE: 1.47). The dashed line represents the linear regression trend.

The study design is presented in Fig. 1. First, we estimated the association between age of onset in a Canadian cohort of 46 unrelated *C9orf72* patients and DNA methylation levels at 7603 common CpG-SNPs available on the 450K BeadChip. Age of onset was significantly associated with DNA methylation levels (q < 0.05) at three CpG-SNPs (rs12763379 on 10q24.2; rs9357140 and rs2143466 on 6p21.3): *P = *9.6 × 10^−6^, *P = *6.0 × 10^−6^ and *P = *1.8 × 10^−5^, respectively (Fig. 2A and Supplementary Table 7). However, rs12763379 in *PYROXD2* was removed from follow-up study because of its overlap with insertion/deletion variations and single tandem repeats precluding reliable genotyping.

### Genetic association study confirmed the association between rs9357140/rs2143466 and age of onset

Multivariate linear regression suggested that rs9357140 genotypes control the gain or loss of DNA methylation at CpG-site cg18698799 (*P < *1.0 × 10^−6^), thereby underlying the association with age of onset: adjusted *P = *2.2 × 10^−5^, B = 7.01 (SE: 1.47) (Fig. 2B). The association remained significant after adjusting for DNA methylation age-acceleration: *P = *2.7 × 10^−4^, B = 6.72 (SE: 1.68). AA-carriers have significantly lower DNA methylation levels compared to AG-carriers (*P = *2.2 × 10^−5^, Mann-Whitney U-test) or GG-carriers (*P = *4.7 × 10^−5^, Mann-Whitney U-test); mean β-value: 0.04 (AA-carriers) versus 0.54 (AG-carriers) versus 0.88 (GG-carriers) (Fig. 2B). Similar results were observed for rs2143466 (Supplementary Fig. 1). The Q-Q plot suggested that there are no other confounders for the association (Supplementary Fig. 2). Both SNPs belong to a strong 124.7 kb LD-block (R^2 ^> 0.8) on chr6:32213638–32338386 containing two overlapping genes: a long non-coding RNA (*LOC101929163*) and *C6orf10*—an uncharacterized testes-specific gene with rs9357140 mapped to intron 9 and rs2143466 mapped to intron 14 (Fig. 2).

**Figure 3:**
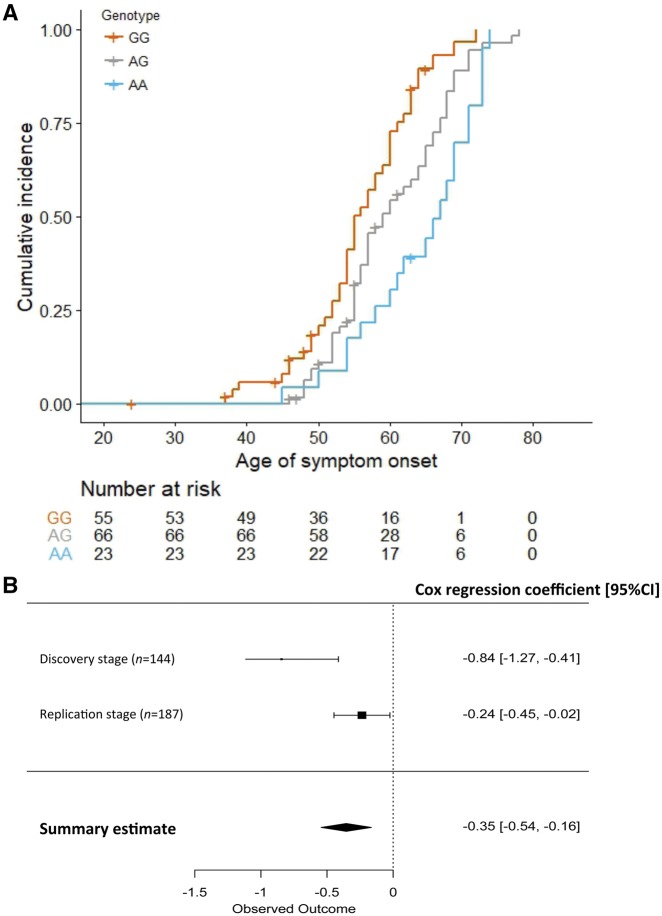
**The association between rs9357140 genotypes and age of onset in *C9orf72* carriers.** (**A**) Kaplan-Meier curve of cumulative incidence of disease onset in the discovery cohort (*n = *144) stratified by rs9357140 genotypes. (**B**) Meta-analysis of the Cox regression coefficient from the discovery cohort (*n = *144) and the replication cohort (*n = *187). The regression coefficient equals logHR.

Next, we enlarged our discovery dataset to 144 carriers by genotyping rs9357140 and rs2143466 in 98 recently collected *C9orf72* carriers, including 101 unrelated symptomatic carriers and 16 families with 21 symptomatic and 22 asymptomatic *C9orf72* carriers (Fig. 1 and Table 1). To obtain the median age of onset for different SNP genotypes, we used the Kaplan-Meier estimate, censoring age of last follow-up for asymptomatic carriers. The median age of onset difference between rs9357140 AA- and GG-carriers was 12 years: 67 years for AA (95% CI: 60–71), 59 years for AG (95% CI: 56–64) and 55 years for GG genotype (95% CI: 54–60) (Fig. 3A). Cox proportional hazard regression analysis also revealed that age of onset in *C9orf72* carriers is significantly associated with rs9357140 genotypes: adjusted *P = *1.1 × 10^−4^, HR = 0.43 (95% CI: 0.28-0.66), suggesting that every A-allele could reduce hazard by 57% (Fig. 3B and Table 2). A similar association with age of onset was also observed for rs2143466: adjusted *P = *1.1 × 10^−4^, HR = 0.43 (95% CI: 0.28–0.68) (Supplementary Fig. 3).

**Table 2:** The Cox proportional hazard regression results for the association between the rs9357140 genotypes and age of onset in the discovery and replication cohorts

	Discovery (*n = *144)	Replication (*n = *187)
HR (95% CI)	0.59 (0.45–0.77)	0.79 (0.64–0.98)
*P*-value for AA versus AG versus GG	0.0001	0.029
Adjusted HR (95% CI)*	0.43 (0.28–0.68)	0.79 (0.64–0.98)
Adjusted *P*-value for AA versus AG versus GG*	0.00011	0.03

*The hazard ratio (HR) and *P*-value was adjusted for sex, rs1990622 genotypes, disease phenotypes and family relationship in the discovery stage. HR was adjusted for sex, rs1990622 genotypes and disease phenotypes in the replication stage.

### The replication study validated the association between rs9357140/rs2143466 and age of onset

In the replication stage (Fig. 1 and Table 1), we obtained genotypes from the IFGC-GWAS (Ferrari *et al.*, 2014) for 10 SNPs tagged by rs9357140 (R^2 ^> 0.9) (Supplementary Table 5) for 187 *C9orf72* patients with a median age of onset of 58 years and interquartile range (IQR) of 51–80 years. Cox proportional hazard regression analysis showed that age of onset was significantly associated with rs9357140: adjusted *P = *0.03, HR = 0.79 (95% CI: 0.64–0.98), suggesting that every A-allele reduced hazard by 21% (Table 2 and Fig. 3). As expected, similar associations with age of onset were observed for rs2143466: adjusted *P = *0.025, HR = 0.79 (95% CI: 0.64–0.97) (Supplementary Fig. 3) and for the other eight SNPs within the LD block listed in Supplementary Table 5 (data not shown).

### Meta-analysis revealed overall effect of rs9357140/rs2143466 on age of onset

We conducted a meta-analysis of logHR in all 331 *C9orf72* carriers using a fixed-effects model and observed that every A-allele of rs9357140 reduced hazard by 30% (pooled HR = 0.70, *P = *0.0003) (Fig. 3B). Again, a similar effect was observed for rs2143466 (pooled HR = 0.70, *P = *0.0002) (Supplementary Fig. 3). The association between age of onset and rs9357140 was also significant in 304 unrelated *C9orf72* patients: adjusted *P = *2.3 × 10^−6^, B = 3.2 (SE: 0.67) (Supplementary Table 1). The median age of onset of rs9357140 AA-carriers was 6 years later than GG-carriers: 62 years (IQR: 57–68) versus 56 years (IQR: 50–62).

### Subgroup analyses of the association between rs9357140 and age of onset

The association between age of onset and rs9357140 was evident in unrelated *C9orf72* patients with either pure ALS (*n = *59; adjusted *P = *0.002, B = 4.97, SE: 1.53) or pure FTD (*n = *174; adjusted *P = *0.0008, B = 2.82, SE: 0.83), but not in patients with FTD-ALS (*n = *71; adjusted *P = *0.125, B = 2.63, SE: 1.69) (Supplementary Table 1 and Supplementary Fig. 4A–C). A similar result was observed for rs2143466 (Supplementary Fig. 4D–F). Notably, we found no significant difference in age of onset among patients affected by pure ALS, pure FTD or FTD-ALS; or ALS/FTD subtypes (bulbar ALS, limb ALS, unspecified ALS, bvFTD, semantic dementia, PNFA, unspecified FTD): *P > *0.05, Kruskal-Wallis test (Supplementary Fig. 5). Multivariate linear regression analysis in different disease subtypes revealed that age of onset was associated with rs9357140 genotypes in limb ALS (*n = *35) and bulbar ALS (*n = *23) under a dominant model (adjusted *P < *0.05), and bvFTD (*n = *157) under a recessive model (adjusted *P < *0.05), but not in FTD-ALS patients (*n = *71) (Supplementary Table 1).

To evaluate if rs9357140 genotypes modify disease phenotypes, we performed a trend analysis using the Cochran–Armitage test to analyse the association between rs9357140 genotypes and *C9orf72* disease phenotypes (ALS versus FTD, ALS versus FTD-ALS, or FTD versus FTD-ALS) under an additive model (AA versus AG versus GG) (Supplementary Table 8); and found no statistically significant results (*P > *0.05).

### Age of onset in *C9orf72* negative patients is associated with rs9357140

**Figure 4:**
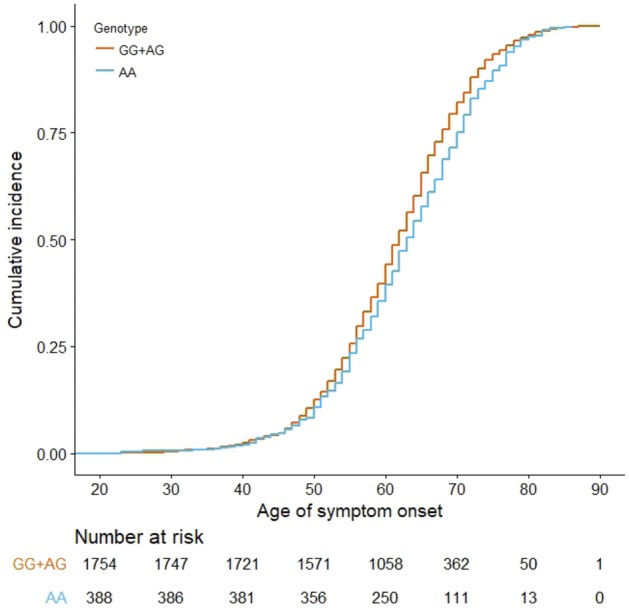
Kaplan-Meier curve of cumulative incidence of disease age of onset in 2142 *C9orf72-*negative patients with FTD stratified by rs9357140 genotype (AA versus GG+AG).

We analysed 2634 *C9orf72*-negative patients with ALS, FTD or FTD-ALS (Supplementary Table 2), and found a significant association between rs9357140 genotypes and age of onset (adjusted *P = *0.007 for recessive model) (Supplementary Table 2). Subgroup analysis detected a significant association only in the largest subgroup of FTD patients (*n = *2142, adjusted *P = *0.01 for recessive model) with a small effect size (B = 1.44, SE: 0.55) (Supplementary Table 2). The association is evident in the bvFTD patients (*n = *1364, adjusted *P = *0.035 for recessive model), but not the other smaller FTD subtypes (Supplementary Table 2). We also observed that age of onset differed significantly among the FTD subtypes (*P < *0.01, Kruskal-Wallis test). Cox proportional hazard regression analysis revealed that the AA-genotype is associated with age of onset [*P = *0.036, adjusted for sex and FTD subtype; HR = 0.94 (95% CI: 0.88–0.99)], suggesting that the AA-genotype could reduce hazard by 6% relative to the GG- and AG-genotypes. Kaplan-Meier estimate analysis revealed that AA-carriers have a slightly later median age of onset (63 years in AA-carriers versus 62 years in GG + AG-carriers) (Fig. 4). A similar association with age of onset was also observed for rs2143466 in the *C9orf72* negative FTD patients: *P = *0.036, adjusted for sex and FTD subtype; HR = 0.94 (95% CI: 0.88–0.99).

### The expression of *HLA-DRB1* and *LOC101929163* is associated with rs9357140

Since rs9357140 and rs2143466 control the loss or gain of CpG-sites and therefore DNA methylation levels (Fig. 2), we hypothesized that CpG-SNPs at the *C6orf10*/*LOC101929163* locus may modulate age of onset by regulating gene expression. We used the public eQTL dataset of 48 types of human tissue (GTEx portal) to analyse if genotypes of the top-significant tagging SNP (rs9357140) are associated with the expression of nearby genes (10 top-significant hits are shown in Supplementary Table 9). Among brain tissues, the A-allele of rs9357140 was associated with reduced expression of the *LOC101929163* (*P = *7.6 × 10^−6^, NES = −0.66 in the nucleus accumbens, part of the basal ganglia; Supplementary Fig. 6A); and *HLA-DRB1*, encoding major histocompatibility complex, class II, DR beta 1 (*P = *4.1 × 10^−6^, NES = −0.42 in the frontal cortex; Supplementary Fig. 6B).

To validate the link between rs9357140 genotypes and *HLA-DRB1* expression, we conducted quantitative RT-PCR using frontal cortex from 25 unrelated ALS cases (Supplementary Fig. 6C). Mann-Whitney U-test confirmed that AA-carriers had significantly lower *HLA-DRB1* expression compared to AG-carriers (*P = *0.001) or GG-carriers (*P = *0.000003). Of note, *C9orf72* expression did not differ among the rs9357140 genotypes (*P > *0.05, Mann-Whitney U-test, Supplementary Fig. 7) and was not correlated with *HLA-DRB1* expression (adjusted *P = *0.23, linear regression).

### Bioinformatics analysis predicted multiple DNase I hypersensitivity sites within the LD-block associated with age of onset

The LD-block associated with age of onset contains 196 known variants tagged by rs9357140 (R^2 ^> 0.8), including five missense substitutions with minor allele frequencies of 0.36–0.38 and conflicting functional predictions by PolyPhen-2 and SIFT (Supplementary Table 10). Since *C6orf10* is expressed mainly in testes, its coding variability is likely not relevant to *C9orf72* pathology. We found no transcriptional factor binding sites mapped to any of the 196 SNPs; however, 12 of these SNPs are located at DNase I hypersensitivity sites; including rs9268000 (78 kb upstream of rs9357140) with a high cluster score of 1000 (Supplementary Table 10). These results suggest that age of onset modifiers of *C9orf72* disease could be associated with DNase I hypersensitivity sites and *HLA-DRB1* expression (250 kb away from the investigated LD-block, Fig. 2).

## Discussion

The current study combined epigenetic and genetic data to detect functional variants associated with age of onset in a large dataset of 331 *C9orf7*2 carriers. A DNA methylation study of CpG-SNPs in the discovery stage enabled prioritizing age of onset modifiers linked to DNA methylation status for further genetic investigation. Such a novel strategy has the advantage of reducing noise from GWAS signals. Indeed, CpG-SNPs could help rank GWAS hits (Gagliano *et al.*, 2016), as they are important elements of methylation quantitative trait loci (Hannon *et al.*, 2016). Our genome-wide DNA methylation analysis of CpG-SNPs followed by a genetic association study of the discovery and replication *C9orf72* cohorts revealed that age of onset is associated with SNPs within a 124.7 kb LD-block tagged by rs9357140. Overall, every A-allele of rs9357140 may reduce hazard by 30% (the median age of onset of AA-carriers was 6 years later than GG-carriers). The genotypes of rs9357140 were also moderately associated with age of onset in *C9orf72*-negative patients although the effect size was small (e.g. the median age of onset in AA-carriers affected by FTD was 1 year later than GG-carriers).

Recently, a key tool for connecting phenotypes to genetic variations has emerged from gene expression studies. Since the locus with significant SNPs may not be the actual disease-related target, *cis*-acting eQTLs can provide a mechanistic link between SNPs and the biological processes they affect (GTEx Consortium *et al.*, 2017). In our study, the minor A-allele of rs9357140 (top-significant SNP within the *C6orf10*/*LOC101929163* locus) is associated with reduced brain expression of *LOC101929163* (in nucleus accumbens) and *HLA-DRB1* (in frontal cortex), while the major G-allele is associated with their increased expression (Supplementary Fig. 6). Future functional studies have to investigate if the non-coding RNA *LOC101929163* is a modulator of *HLA-DRB1* expression (e.g. affecting transcriptional factors relevant to *HLA-DRB1*). The major histocompatibility complex class II protein HLA-DR is implicated in neurodegenerative diseases as a marker of activated microglia (Walker and Lue, 2015) and is important in initiating immune responses by presenting peptides derived not only from exogenous but also endogenous proteins, such as peptides resulting from autophagy of intracellular proteins by lysosomes (Dengjel *et al.*, 2005).

Our results support the notion that microglial/autophagy pathways play key roles in modulating *C9orf72* disease, the pathogenesis of which might involve both gain and loss of function mechanisms (Hardy and Rogaeva, 2014). Normal function of C9orf72 is essential for the lysosome/autophagosome pathway and immune responses in macrophages or microglia (O’Rourke *et al.*, 2016; Shi *et al.*, 2018). For instance, transcriptome and histologic analyses of *C9orf72* carriers support the idea that decreased *C9orf72* expression leads to altered microglial function and neuroinflammation (O’Rourke *et al.*, 2016), while increased C9orf72 levels could be neuroprotective (McGoldrick *et al.*, 2018; Shi *et al.*, 2018). It is important to investigate if rs9357140 GG-carriers, which have an earlier age of onset and upregulated *HLA-DRB1*, are in a more pro-inflammatory state (e.g. by microglia) than AA-carriers.

Our survey of the literature and the GWAS catalogue database (https://www.ebi.ac.uk/gwas/) revealed that SNPs within or close to the *C6orf10*/*LOC101929163* locus (Supplementary Fig. 8) are associated with autoimmune disorders (multiple sclerosis, rheumatoid arthritis, systemic sclerosis, Grave’s disease and asthma), as well as neurodegenerative diseases (FTD, Parkinson’s disease and Alzheimer’s disease) (Lambert *et al.*, 2013; Ferrari *et al.*, 2014; Lu *et al.*, 2017), highlighting the role of the immune system in neurodegeneration (Supplementary Table 11). Notably, several dementia genes are linked to microglia/immune function (e.g. *TREM2* and *CD33*) (Lambert *et al.*, 2013). Our study of *C9orf72*-negative patients suggests that the *C6orf10*/*LOC101929163* locus could be a modest age of onset modifier for the general population of FTD patients. Intriguingly, another two SNPs (rs9268877 and rs9268856) near this locus have been reported in a case-control GWAS as modifiers of FTD risk (Ferrari *et al.*, 2014). Of note, rs9357140 is not in LD with rs9268877 and rs9268856 representing an independent association signal (Supplementary Table 11), yet the mechanism behind the association with age of onset or disease risk could be similarly pointing to the functional significance of the *HLA-DRA*/*HLA-DRB5* locus.

Notably, age of onset estimation is more objective for ALS (self-reported) than FTD (reported by family members) (Pottier *et al.*, 2018). Hence, the less significant result in the replication *C9orf72* cohort enriched in FTD patients (72.7% versus 27.8% in the discovery stage) may be explained by a less accurate age of onset estimation (Supplementary Table 12). In addition, the subgroup analysis could be further complicated by the less accurate estimation of age of onset for the complex FTD-ALS phenotype and reduced statistical power for the smaller subgroup.

One of the limitations of our study is the lack of unified deep phenotyping for each patient and healthy control data, however our findings set the basis for future research (e.g. aimed at investigating the link between CpG-SNPs and disease phenotype, risk, progression or severity). Another limitation is the absence of information on the expansions size in our study participants, because *C9orf72* genotyping was done by repeat-primed PCR. This is of note, since repeat length examined by Southern blot was inversely correlated with age of onset (van Blitterswijk *et al.*, 2013), and the clinical data support disease anticipation in *C9orf72* families, which is evident by an earlier age of onset across successive generations (van Blitterswijk *et al.*, 2013; Xi *et al.*, 2015*a*; Van Mossevelde *et al.*, 2017). However, *C9orf72* repeat expansions are difficult to size accurately by Southern blot because of their large size (up to several thousand repeats) and somatic mosaicism masking the true length of the expansion (Xi *et al.*, 2015*a*; McGoldrick *et al.*, 2018). It would also be important to understand the genetic-epigenetic links across human tissues relevant to neurodegenerative disorders, since DNA methylation changes reflect the complex interactions between genes, environmental factors, and ageing (Zhang *et al.*, 2016).

Our findings suggest that CpG-SNPs at the *C6orf10*/*LOC101929163* locus might modify age of onset in *C9orf72* carriers belonging to the entire ALS-FTD spectrum by controlling DNA methylation and gene expression (e.g. *HLA-DRB1*). CpG-SNPs at the *C6orf10*/*LOC101929163* locus might also be age of onset modifiers for general FTD patients to a lesser extent. Understanding the functional mechanisms of the *C6orf10*/*LOC101929163*/*HLA-DRB1* pathway (e.g. to investigate if the non-coding RNA *LOC101929163* is a modulator of *HLA-DRB1* expression) might prove critical for identifying biomarkers and/or designing drugs to modify age of onset in *C9orf72* driven disease. Finally, the detected CpG-SNPs could be used to better predict age of onset in *C9orf72* asymptomatic carriers in preventive clinical trials (e.g. based on the Genetic Frontotemporal dementia Initiative study) (Rohrer *et al.*, 2015), for designing conditional and/or modifiers studies in the sporadic FTLD spectrum, such as based on IFGC related projects (https://ifgcsite.wordpress.com/) and for genetic counselling.

## Supplementary Material

Supplementary Figures and Tables

Supplementary Materials

## Abbreviations

ALS: amyotrophic lateral sclerosis
FTD: frontotemporal dementia
GWAS: genome wide association study
LD: linkage disequilibrium
SNP: single nucleotide polymorphism

## Appendix 1

We acknowledge the following members of the International FTD-Genomics Consortium (https://ifgcsite.wordpress.com/) for their contribution of genotyping and clinical data of FTD/FTD-ALS patients (funding, as well as full location and affiliations details can be found in the Supplementary material): Raffaele Ferrari, Dena G. Hernandez, Michael A. Nalls, Jonathan D. Rohrer, Adaikalavan Ramasamy, John B. J. Kwok, Carol Dobson-Stone, William S. Brooks, Peter R. Schofield, Glenda M. Halliday, John R. Hodges, Olivier Piguet, Lauren Bartley, Elizabeth Thompson, Isabel Hernández, Agustín Ruiz, Mercè Boada, Barbara Borroni, Alessandro Padovani, Carlos Cruchaga, Nigel J. Cairns, Luisa Benussi, Giuliano Binetti, Roberta Ghidoni, Gianluigi Forloni, Diego Albani, Daniela Galimberti, Chiara Fenoglio, Maria Serpente, Elio Scarpini, Jordi Clarimón, Alberto Lleó, Rafael Blesa; Maria Landqvist Waldö, Karin Nilsson, Christer Nilsson, Ian R. A. Mackenzie, Ging-Yuek R. Hsiung, David M. A. Mann, Jordan Grafman, Christopher M. Morris, Johannes Attems, Timothy D. Griffiths, Ian G. McKeith, Alan J. Thomas, Pietro Pietrini, Edward D. Huey, Eric M. Wassermann, Atik Baborie, Evelyn Jaros, Michael C. Tierney, Pau Pastor, Cristina Razquin, Sara Ortega-Cubero, Elena Alonso, Robert Perneczky, Janine Diehl-Schmid, Panagiotis Alexopoulos, Alexander Kurz, Innocenzo Rainero, Elisa Rubino, Lorenzo Pinessi, Ekaterina Rogaeva, Peter St George-Hyslop, Giacomina Rossi, Fabrizio Tagliavini, Giorgio Giaccone, James B. Rowe, Johannes C. M. Schlachetzki, James Uphill, John Collinge, Simon Mead, Adrian Danek, Vivianna M. Van Deerlin, Murray Grossman, John Q. Trojanowski, Julie van der Zee, Christine Van Broeckhoven, Stefano F. Cappa, Isabelle Leber, Didier Hannequin, Véronique Golfier, Martine Vercelletto, Alexis Brice, Benedetta Nacmias, Sandro Sorbi, Silvia Bagnoli, Irene Piaceri, Jørgen E. Nielsen, Lena E. Hjermind, Matthias Riemenschneider, Manuel Mayhaus, Bernd Ibach, Gilles Gasparoni, Sabrina Pichler, Wei Gu, Martin N Rossor, Nick C. Fox, Jason D. Warren, Maria Grazia Spillantini, Huw R. Morris, Patrizia Rizzu, Peter Heutink, Julie S. Snowden, Sara Rollinson, Anna Richardson, Alexander Gerhard, Amalia C. Bruni, Raffaele Maletta, Francesca Frangipane, Chiara Cupidi, Livia Bernardi, Maria Anfossi, Maura Gallo, Maria Elena Conidi, Nicoletta Smirne, Rosa Rademakers, Matt Baker, Dennis W. Dickson, Neill R. Graff-Radford, Ronald C. Petersen, David Knopman, Keith A. Josephs, Bradley F. Boeve, Joseph E. Parisi, William W. Seeley, Bruce L. Miller, Anna M. Karydas, Howard Rosen, John C. van Swieten, Elise G. P. Dopper, Harro Seelaar, Yolande A. L. Pijnenburg, Philip Scheltens, Giancarlo Logroscino, Rosa Capozzo, Valeria Novelli, Annibale A. Puca, Massimo Franceschi, Alfredo Postiglione, Graziella Milan, Paolo Sorrentino, Mark Kristiansen, Huei-Hsin Chiang, Caroline Graff, Florence Pasquier, Adeline Rollin, Vincent Deramecourt, Thibaud Lebouvier, Dimitrios Kapogiannis, Luigi Ferrucci, Stuart Pickering-Brown, Andrew B. Singleton, John Hardy, Parastoo Momeni.
