# Supplementary material for: A C6orf10/LOC101929163 locus is associated with age of onset in C9orf72 carriers
Source: Brain. 2018 Sep 25;141(10):2895–907. doi: 10.1093/brain/awy238 (PMC6158742; doi:10.1093/brain/awy238)
Supplement: Supplementary Figures and Tables [file awy238_supplementary_figures-tables.pdf]

**Supplementary materials**

| **Table of contents** | **Content** |
|---|---|
| Supplementary Table 1 | Clinical characteristics and multivariate linear regression results of disease subtypes in unrelated *C9orf72* patients. |
| Supplementary Table 2 | Clinical characteristics and multivariate linear regression results of disease subtypes in unrelated *C9orf72* negative patients. |
| Supplementary Table 3 | Sample characteristics of the ALS cases whose frontal cortex tissues were used in the gene expression experiments. |
| Supplementary Table 4 | PCR primers used to obtain genotypes for rs9357140, rs2143466 and rs1990622. |
| Supplementary Table 5 | The variants genotyped or imputed for the replication cohort. |
| Supplementary Table 6 | The results of testing endogenous gene expression in nine frontal cortex tissues (n=3 per rs9357140 genotype) using Normfinder. |
| Supplementary Table 7 | The association between DNA methylation of CpG-SNPs and age of onset in 46 *C9orf72* patients (10 top-significant CpGs are listed). |
| Supplementary Table 8 | The distribution of rs9357140 genotypes in *C9orf72* ALS, FTD and FTD-ALS patients. |
| Supplementary Table 9 | eQTL analysis using the GTeX database revealed significant changes in gene expression associated with rs9357140 genotypes in different tissues (10 top-significant hits are listed). |
| Supplementary Table 10 | Coding and non-coding effects of the 196 variants in the *C6orf10/LOC101929163* LD-block (chr6: 32213638 – 32338386) associated with age of onset in *C9orf72* carriers. |
| Supplementary Table 11 | The SNPs located within or close to the *C6orf10/LOC101929163* locus (chr6: 32213638–32338386) reported to be associated with neurodegenerative or autoimmune diseases. |
| Supplementary Table 12 | The distribution of phenotypes for C9orf72 carriers in the discovery and replication stages. |
| Supplementary Figure 1 | Genotypes of rs2143466 in the discovery cohort are significantly associated with DNA methylation status ($p<1.0\times10^{-6}$, B=-0.25) and AO ($p=7.0\times10^{-6}$ adjusted for sex and *TMEM106B* genotypes, B=7.1). |
| Supplementary Figure 2 | A Q-Q plot of the genome-wide DNA methylation analysis of CpG-SNPs. |
| Supplementary Figure 3 | The result of the Cox regression coefficient meta-analysis of rs2143466 for the discovery (n=141) and replication (n=187) cohorts. |
| Supplementary Figure 4 | Box-Scatter plots representing the association between rs9357140 or rs2143466 genotypes |

| | |
|---|---|
| | and age of onset in *C9orf72* patients with ALS, FTD and FTD-ALS. |
| Supplementary Figure 5 | Scatter plot of AO in disease subtypes of unrelated *C9orf72* patients (n=304 in total). Mean AO and standard error bar of each group are presented. |
| Supplementary Figure 6 | Results of the association study of rs9357140 using GTEx eQTL data (Ref=G-allele, Alt=A-allele) and quantitative RT-PCR. |
| Supplementary Figure 7 | Genotypes of rs9357140 are not associated with *C9orf72* expression (p>0.05, Mann Whitney U test) in frontal cortex tissues from 25 ALS cases. Expression *C9orf72* and *HLA-DRB1* are not significantly correlated with each other (p=0.23, multivariate linear regression, adjusted for sex). |
| Supplementary Figure 8 | LD-plot with the location of the genetic variations (rs547077, rs9405090, rs9357140, rs2143466, and rs9268368) significantly associated with age of onset in *C9orf72* patients (in red font), as well as nearby top-significant variants (rs404860, rs443198, rs9296015, rs6910071, rs2395148, rs3129934, rs2273017, rs3129871, rs3129882, rs3135388, rs9268856, and rs9268877) reported to be linked to neurodegenerative (blue font) or autoimmune (green font) diseases. |
| Appendix | Acknowledgments for the International FTD-Genomics Consortium (IFGC) |

**Supplementary Table 1.** Clinical characteristics and multivariate linear regression results of disease subtypes in unrelated *C9orf72* patients. Additive model: AA vs AG vs GG; dominant model: AA or AG vs GG; recessive model: AA vs AG or GG. P-values <0.05 are presented in bold font.

| | | **Combined *C9orf72* patients** | **Disease phenotypes** | | | **ALS subtypes** | | | **FTD subtypes** | | | |
|---|---|---|---|---|---|---|---|---|---|---|---|---|
| | | | **Combined ALS** | **Combined FTD** | **Combined FTD-ALS** | **Bulbar ALS** | **Limb ALS** | **UALS** | **bvFTD** | **PNFA** | **SD** | **UFTD** |
| Number of cases (%) | | 304 (100%) | 59 (19.4%) | 174 (57.2%) | 71 (23.3%) | 23 (7.5%) | 35 (12.8%) | 1 (0.3%) | 157 (51.6%) | 7 (2.3%) | 7 (2.3%) | 3 (1%) |
| Sex (Male, n, percentage) | | 167 (54.9%) | 27 (45.7%) | 100 (57.5%) | 40 (56.3%) | 8 (34.8%) | 18 (51.4%) | 1 (100%) | 92 (58.6) | 5 (71%) | 3 (42.9%) | 0 (0%) |
| AO (years, median, IQR) | | 58 (52-64) | 58 (55-67.5) | 58 (51.3-63) | 57 (52-64) | 60 (57-68) | 57 (54-67) | NA | 58 (51-63) | 60 (58-62.5) | 58 (50-62.5) | 62 (56.5-65) |
| AO (years, mean, range) | | 59 (34-80) | 59.5 (37-78) | 57.4 (34-80) | 57.9 (38-73) | 61.4 (51-73) | 58.4 (37-78) | NA | 57.2 (34-77) | 60.9 (56-69) | 58.7 (48-80) | 60.3 (51-68) |
| Additive Model | adjusted p* | **0.0000023** | **0.002** | **0.0008** | 0.125 | 0.07 | **0.015** | NA | **0.0043** | NA | NA | NA |
| | $r^2$ | 0.077 | 0.19 | 0.057 | 0.06 | 0.04 | 0.28 | NA | 0.078 | NA | NA | NA |
| | B (SE) | 3.2 (0.67) | 4.97 (1.53) | 2.82 (0.8) | 2.63 (1.69) | 3.77 (2) | 5.9 (2.3) | NA | 2.52 (0.87) | NA | NA | NA |
| Dominant Model | adjusted p* | **0.00003** | **0.0002** | **0.04** | 0.14 | **0.04** | **0.001** | NA | 0.07 | NA | NA | NA |
| | $r^2$ | 0.06 | 0.26 | 0.02 | 0.02 | 0.09 | 0.3 | NA | 0.03 | NA | NA | NA |
| | B (SE) | 1.44 (0.95) | 8.5 (2.1) | 2.6 (1.3) | 3.4 (2.3) | 5.9 (2.7) | 11.1 (3) | NA | 2.4 (1.3) | NA | NA | NA |
| Recessive Model | adjusted p* | **0.0005** | 0.3 | **0.0004** | 0.4 | 0.47 | 0.6 | NA | **0.001** | NA | NA | NA |
| | $r^2$ | 0.04 | 0.06 | 0.07 | 0.03 | 0.4 | 0.04 | NA | 0.07 | NA | NA | NA |
| | B (SE) | 4.5 (1.3) | 3.3 (3.2) | 5.7 (1.6) | 2.6 (3.2) | 3.3 (4.4) | 2.5 (4.6) | NA | 5.4 (1.7) | NA | NA | NA |

*p-values adjusted for sex and rs1990622 genotypes; NA = not applicable, UALS = unspecified ALS; UFTD= unspecified FTD; bvFTD = behavioural frontotemporal dementia, SD = semantic dementia, PNFA = progressive nonfluent aphasia.

**Supplementary Table 2.** Clinical characteristics and multivariate linear regression results of disease subtypes in unrelated *C9orf72* negative patients. Additive model: AA vs AG vs GG; dominant model: AA or AG vs GG; recessive model: AA vs AG or GG. P-values <0.05 are presented in bold font.

| | | ***C9orf72* negative patients** | **Disease phenotypes** | | | **FTD subtypes** | | | |
|---|---|---|---|---|---|---|---|---|---|
| | | | **ALS** | **FTD** | **FTD-ALS** | **bvFTD** | **PNFA** | **SD** | **UFTD** |
| Number of cases (%) | | 2634 (100%) | 328 (12.5%) | 2142 (81.3%) | 164 (6.2%) | 1364 (51.8%) | 326 (12.4%) | 359 (13.6%) | 93 (3.5%) |
| Sex (Male, n, percentage) | | 1441 (54.7%) | 196 (59.8%) | 1136 (53%) | 109 (66.5%) | 727 (53.3%) | 156 (47.9%) | 205 (57.1%) | 48 (51.6%) |
| AO (years, median, IQR) | | 62 (55-69) | 60 (51-69) | 62 (55-65) | 61.5 (56-68) | 62 (56-66.5) | 65 (58-71) | 61.5 (55-68) | 64 (56-70) |
| AO (years, mean, range) | | 61.5 (23-90) | 59.5 (24-87) | 61.8 (23-90) | 61.6 (31-89) | 61.8 (41-85) | 64.5 (41-86) | 61.1 (23-90) | 63 (31-85) |
| Additive Model | adjusted p* | **0.03** | 0.39 | **0.03** | 0.47 | **0.04** | 0.1 | 0.32 | 0.06 |
| | $r^2$ | 0.007 | 0.002 | 0.01 | 0.008 | 0.013 | 0.019 | 0.003 | 0.022 |
| | B (SE) | 0.61 (0.28) | 0.85 (0.97) | 0.66 (0.3) | -0.8 (1.2) | 0.81 (0.4) | 1.1 (0.68) | -0.62 (0.63) | 3.1 (1.6) |
| Dominant Model | adjusted p* | 0.33 | 0.75 | 0.24 | 0.38 | 0.19 | 0.07 | **0.032** | 0.1 |
| | $r^2$ | 0.006 | 0.0003 | 0.01 | 0.005 | 0.01 | 0.02 | 0.009 | 0.01 |
| | B (SE) | 0.41 (0.42) | 0.45 (1.4) | 0.52 (0.45) | -1.3 (1.5) | 0.78 (0.6) | 1.83 (1) | -1.9 (0.9) | 3.9 (2.3) |
| Recessive Model | adjusted p* | **0.007** | 0.23 | **0.01** | 0.9 | **0.035** | 0.47 | 0.3 | 0.16 |
| | $r^2$ | 0.009 | 0.004 | 0.01 | 0.0002 | 0.013 | 0.012 | 0.004 | 0.003 |
| | B (SE) | 1.41 (0.52) | 2.2 (1.8) | 1.4 (0.55) | -0.3 (2.4) | 1.49 (0.7) | 0.92 (1.28) | 1.2 (1.2) | 4.1 (2.9) |

*p-values adjusted for sex.

**Supplementary Table 3.** Sample characteristics of the ALS cases whose frontal cortex tissues were used in the gene expression experiments.

| | |
|---|---|
| **Total number** | 25 |
| **Sex (number of men, %)** | 15 (60%) |
| **Age of death (mean, SD) in years** | 64.24 (11.05) |
| **Age of death (median, IQR) in years** | 65 (59-72) |

IQR = interquartile range

**Supplementary Table 4.** PCR primers used to obtain genotypes for rs9357140, rs2143466 and rs1990622.

| Primer name | Primer sequence |
|---|---|
| rs9357140 forward | 5'-GGTCCTAGGTGGCTATGTGG-3' |
| rs9357140 reverse | 5'-GAAAGCCATCTTCCAGGTGT-3' |
| rs2143466 forward | 5'-TGAAAAGTCCATGCCCTACC-3' |
| rs2143466 reverse | 5'-TTCACTTGCCCTATCCCAAC-3' |
| rs1990622 forward | 5'- GCATTGTGTTTGATTGTAGGGG-3' |
| rs1990622 reverse | 5'- ACTCCAGGACTTATGTGGCC-3' |

**Supplementary Table 5.** The variants genotyped or imputed for the replication cohort.

| CHR | POS | rs# | REF | ALT | Genotyped | Imputed | Imputation score |
|---|---|---|---|---|---|---|---|
| **FTD-GWAS Phase I (n=150)** | | | | | | | |
| **CHR** | **POS** | **rs#** | **REF** | **ALT** | **Genotyped** | **Imputed** | **Imputation score** |
| 6 | 32282033 | rs547261 | G | A | | yes | 0.99 |
| 6 | 32289318 | rs547077 | T | C | yes | | |
| 6 | 32290954 | rs485774 | A | G | | yes | 0.99 |
| 6 | 32297337 | rs9368713 | T | C | yes | | |
| 6 | 32298372 | rs9405090 | A | G | yes | | |
| 6 | 32301514 | rs9357140 | G | A | | yes | 0.99 |
| 6 | 32307382 | rs1033500 | G | A | | yes | 0.99 |
| 6 | 32333955 | rs9268368 | T | C | yes | | |
| 6 | 32336586 | rs9268384 | A | G | yes | | |
| 6 | 32309323 | rs2143466 | C | T | | yes | 0.99 |
| 7 | 12283787 | rs1990622 | A | G | | yes | 0.99 |
| **FTD-GWAS Phase II (n=37)** | | | | | | | |
| **CHR** | **POS** | **rs#** | **REF** | **ALT** | **Genotyped** | **Imputed** | **Imputation score** |
| 6 | 32282033 | rs547261 | G | A | yes | | |
| 6 | 32289318 | rs547077 | T | C | yes | | |
| 6 | 32290954 | rs485774 | A | G | | yes | 0.99 |
| 6 | 32297337 | rs9368713 | T | C | | yes | 0.99 |
| 6 | 32298372 | rs9405090 | A | G | yes | | |
| 6 | 32301514 | rs9357140 | G | A | | yes | 0.99 |
| 6 | 32307382 | rs1033500 | G | A | yes | | |
| 6 | 32333955 | rs9268368 | T | C | yes | | |
| 6 | 32336586 | rs9268384 | A | G | | yes | 0.99 |
| 6 | 32309323 | rs2143466 | C | T | | yes | 1 |
| 7 | 12283787 | rs1990622 | A | G | | yes | 0.43 |

**Supplementary Table 6.** Results of testing endogenous gene expression in nine frontal cortex tissues (n=3 per rs9357140 genotype) using Normfinder. Lower stability values represent less variability.

| Gene name | Stability value |
|---|---|
| *UBC* | 0.359 |
| *HPRT1* | 0.379 |
| *B2M* | 0.049 |
| *RPLP0* | 0.099 |

**Supplementary Table 7.** The association between DNA methylation of CpG-SNPs and age of onset in 46 *C9orf72* patients (10 top-significant CpGs are listed).

| CpG ID | p-value | q-value | Rs# | Gene |
|---|---|---|---|---|
| **cg18698799** | 6.0E-06 | 0.035 | rs9357140 | *C6orf10* |
| **cg26690318** | 9.6E-06 | 0.035 | rs12763379 | *PYROXD2* |
| **cg10528537** | 1.8E-05 | 0.045 | rs2143466 | *C6orf10* |
| **cg04541421** | 0.00046 | 0.595 | rs11647 | *C2orf74* |
| **cg01429471** | 0.00058 | 0.590 | rs56142004 | |
| **cg00892703** | 0.00062 | 0.590 | rs62068681 | *CPNE7* |
| **cg15102777** | 0.00063 | 0.590 | rs17153581 | *BCCIP* |
| **cg08324400** | 0.00064 | 0.590 | rs12652492 | |
| **cg01733958** | 0.00075 | 0.615 | rs1573732 | *TMEM8A* |
| **cg27443567** | 0.00085 | 0.630 | rs2297186 | *PCID2* |

**Supplementary Table 8.** The distribution of rs9357140 genotypes in *C9orf72* ALS, FTD and FTD-ALS patients.

| | AA (n, %) | AG (n, %) | GG (n, %) | Total number | Trend analysis | p-value |
|---|---|---|---|---|---|---|
| **ALS** | 9 (15.3%) | 27 (45.8%) | 23 (39%) | 59 | ALS vs FTD | 0.98 |
| **FTD-ALS** | 8 (11.3%) | 45 (63.3%) | 18 (25.4%) | 71 | ALS vs FTD-ALS | 0.39 |
| **FTD** | 32 (18.4%) | 69 (39.7%) | 73 (41.9%) | 174 | FTD vs FTD-ALS | 0.33 |

**Supplementary Table 9.** eQTL analysis using the GTeX database revealed significant changes in gene expression associated with rs9357140 genotypes in different tissues (10 top-significant hits are listed).

| Gencode Id | Gene Symbol | P-Value | NES | Tissue |
|---|---|---|---|---|
| ENSG00000225914.1 | *LOC101929163* | 7.6E-09 | -0.66 | Brain - Nucleus accumbens (basal ganglia) |
| ENSG00000196126.6 | *HLA-DRB1* | 4.1E-06 | -0.42 | Brain – Frontal cortex |
| ENSG00000196126.6 | *HLA-DRB1* | 2.3E-05 | -0.3 | Testis |
| ENSG00000196126.6 | *HLA-DRB1* | 3.4E-06 | -0.19 | Skin - Sun Exposed (Lower leg) |
| ENSG00000196126.6 | *HLA-DRB1* | 5.0E-05 | -0.17 | Adipose - Subcutaneous |
| ENSG00000204308.6 | *RNF5* | 2.6E-07 | 0.12 | Muscle - Skeletal |
| ENSG00000204296.7 | *C6orf10* | 5.3E-08 | 0.13 | Testis |
| ENSG00000213676.6 | *ATF6B* | 1.1E-05 | 0.13 | Muscle - Skeletal |
| ENSG00000213676.6 | *ATF6B* | 2.4E-05 | 0.15 | Adipose - Subcutaneous |
| ENSG00000204314.6 | *PRRT1* | 2.6E-06 | 0.17 | Esophagus - Mucosa |

**Supplementary Table 10.** Coding and non-coding effects of 196 variants in the *C6orf10/LOC101929163* LD-block (chr6: 32213638 – 32338386) associated with age of onset in *C9orf72* carriers. The 10 SNPs (including rs9357140) investigated in the current study are in red. NA = not applicable. See the pdf file (Supplementary Table 10.pdf).

Supplementary Table 10.Coding and non-coding effects of 196 variants in the C6orf10/LOC101929163 LD-block (chr6: 32213638 – 32338386) associated with age of onset in C9orf72 carriers

| RS_Number | Position | Alleles | | Minor allele frequencies | Distance | Dprime | $R^2$ | Correlated Alleles | Function | Amino acid change | Polyphen | SIFT | CpG-SNP | DNaseI hyperactivity site | Items (total 125) | Cluster score (total 1000) | Transcriptional factor binding sites |
|---|---|---|---|---|---|---|---|---|---|---|---|---|---|---|---|---|---|
| rs9267956 | 32213638 | A | G | 0.3618 | -87876 | 0.9738 | 0.9124 | G=A,A=G | NA | | | | yes | chr6:32213606-32213775 | 2 | 225 | no |
| rs1559876 | 32215769 | G | C | 0.3588 | -85745 | 0.9868 | 0.9249 | G=G,A=C | NA | | | | yes | | | | no |
| rs9267996 | 32221934 | A | G | 0.3588 | -79580 | 0.9868 | 0.9249 | G=A,A=G | NA | | | | no | | | | no |
| rs9268000 | 32223531 | A | C | 0.34 | -77983 | 0.9954 | 0.866 | G=A,A=C | NA | | | | no | chr6:32222966-32223535 | 114 | 1000 | no |
| rs147193449 | 32227488 | A | G | 0.3718 | -74026 | 1 | 0.9958 | G=A,A=G | NA | | | | no | | | | no |
| rs144338179 | 32227912 | G | - | 0.3718 | -73602 | 1 | 0.9958 | G=G,A=- | NA | | | | no | | | | no |
| rs9268045 | 32229655 | C | T | 0.3718 | -71859 | 1 | 0.9958 | G=C,A=T | NA | | | | no | | | | no |
| rs4959091 | 32231014 | G | A | 0.3718 | -70500 | 1 | 0.9958 | G=G,A=A | NA | | | | yes | | | | no |
| rs2395114 | 32233547 | A | G | 0.3718 | -67967 | 1 | 0.9958 | G=A,A=G | NA | | | | no | | | | no |
| rs9268082 | 32234953 | G | T | 0.3718 | -66561 | 1 | 0.9958 | G=G,A=T | NA | | | | no | chr6:32234786-32235115 | 3 | 371 | no |
| rs9268086 | 32238266 | C | T | 0.3718 | -63248 | 1 | 0.9958 | G=C,A=T | NA | | | | yes | | | | no |
| rs2395115 | 32238802 | T | C | 0.3718 | -62712 | 1 | 0.9958 | G=T,A=C | NA | | | | no | | | | no |
| rs2022539 | 32239558 | A | C | 0.3718 | -61956 | 1 | 0.9958 | G=A,A=C | NA | | | | no | | | | no |
| rs9268113 | 32248166 | G | C | 0.3688 | -53348 | 0.9829 | 0.9578 | G=G,A=C | NA | | | | yes | | | | no |
| rs9268115 | 32248364 | C | T | 0.3748 | -53150 | 1 | 0.9831 | G=C,A=T | NA | | | | yes | | | | no |
| rs7454557 | 32252274 | A | G | 0.3718 | -49240 | 1 | 0.9958 | G=A,A=G | NA | | | | yes | | | | no |
| rs9268129 | 32254225 | G | C | 0.3658 | -47289 | 0.9957 | 0.9704 | G=G,A=C | NA | | | | yes | | | | no |
| rs9268141 | 32256132 | G | A | 0.3658 | -45382 | 0.9957 | 0.9704 | G=G,A=A | NA | | | | yes | | | | no |
| rs4713518 | 32257337 | A | G | 0.3718 | -44177 | 1 | 0.9958 | G=A,A=G | NA | | | | yes | | | | no |
| rs482194 | 32260559 | A | G | 0.3718 | -40955 | 1 | 0.9958 | G=A,A=G | NA | | | | no | | | | no |
| rs560505 | 32261771 | A | G | 0.3658 | -39743 | 0.9957 | 0.9704 | G=A,A=G | missense | p.Ser227Pro | benign | deleterious | no | | | | no |
| rs518374 | 32265710 | A | C | 0.3718 | -35804 | 1 | 0.9958 | G=A,A=C | NA | | | | no | | | | no |
| rs565571 | 32266535 | G | A | 0.3658 | -34979 | 0.9957 | 0.9704 | G=G,A=A | NA | | | | yes | chr6:32266401-32266970 | 44 | 560 | no |
| rs537757 | 32268501 | A | T | 0.3718 | -33013 | 1 | 0.9958 | G=A,A=T | NA | | | | no | | | | no |
| rs533876 | 32268980 | A | G | 0.3718 | -32534 | 1 | 0.9958 | G=A,A=G | NA | | | | no | | | | no |
| rs477005 | 32270500 | A | G | 0.3658 | -31014 | 0.9957 | 0.9704 | G=A,A=G | NA | | | | no | | | | no |
| rs544358 | 32273158 | G | C | 0.3718 | -28356 | 1 | 0.9958 | G=G,A=C | NA | | | | no | | | | no |
| rs540926 | 32276169 | A | G | 0.3718 | -25345 | 1 | 0.9958 | G=A,A=G | NA | | | | no | | | | no |
| rs484302 | 32276300 | G | A | 0.3718 | -25214 | 1 | 0.9958 | G=G,A=A | NA | | | | yes | | | | no |
| rs1265776 | 32276502 | A | G | 0.3718 | -25012 | 1 | 0.9958 | G=A,A=G | NA | | | | yes | | | | no |
| rs502626 | 32278266 | A | G | 0.3718 | -23248 | 1 | 0.9958 | G=A,A=G | NA | | | | no | | | | no |
| **rs547261** | 32282033 | G | A | 0.3718 | -19481 | 1 | 0.9958 | G=G,A=A | NA | | | | no | | | | no |
| rs143081527 | 32282622 | ACTCTAG | - | 0.3718 | -18892 | 1 | 0.9958 | G=TTTGACAACT | NA | | | | no | | | | no |
| rs495140 | 32284423 | A | T | 0.3718 | -17091 | 1 | 0.9958 | G=A,A=T | NA | | | | no | chr6:32284261-32284475 | 2 | 200 | no |
| rs567828 | 32284950 | T | A | 0.3718 | -16564 | 1 | 0.9958 | G=T,A=A | NA | | | | no | | | | no |
| rs531094 | 32286102 | T | A | 0.3718 | -15412 | 1 | 0.9958 | G=T,A=A | NA | | | | no | | | | no |
| rs557539 | 32286680 | G | A | 0.3787 | -14834 | 1 | 0.9666 | G=G,A=A | NA | | | | no | | | | no |
| rs560324 | 32286984 | T | G | 0.3787 | -14530 | 1 | 0.9666 | G=T,A=G | NA | | | | no | | | | no |
| rs2746115 | 32287329 | C | T | 0.3757 | -14185 | 1 | 0.979 | G=C,A=T | NA | | | | no | chr6:32287266-32287415 | 1 | 177 | no |
| rs580921 | 32287496 | A | G | 0.3509 | -14018 | 0.9955 | 0.9092 | G=A,A=G | NA | | | | yes | | | | no |
| rs575765 | 32288041 | T | C | 0.3718 | -13473 | 1 | 0.9958 | G=T,A=C | NA | | | | no | | | | no |
| rs574710 | 32288190 | T | C | 0.3787 | -13324 | 1 | 0.9666 | G=T,A=C | NA | | | | yes | | | | no |
| rs539703 | 32288462 | A | C | 0.3718 | -13052 | 1 | 0.9958 | G=A,A=C | NA | | | | yes | | | | no |
| rs540639 | 32288564 | T | G | 0.3787 | -12950 | 1 | 0.9666 | G=T,A=G | NA | | | | yes | | | | no |
| rs542525 | 32288805 | T | C | 0.3787 | -12709 | 1 | 0.9666 | G=T,A=C | NA | | | | yes | | | | no |
| rs544100 | 32288932 | T | A | 0.3718 | -12582 | 1 | 0.9958 | G=T,A=A | NA | | | | no | | | | no |
| rs546857 | 32289240 | C | T | 0.3658 | -12274 | 0.9957 | 0.9704 | G=C,A=T | NA | | | | no | | | | no |
| **rs547077** | 32289318 | T | C | 0.3787 | -12196 | 1 | 0.9666 | G=T,A=C | NA | | | | no | | | | no |
| rs572730 | 32289764 | C | T | 0.3718 | -11750 | 1 | 0.9958 | G=C,A=T | NA | | | | no | | | | no |
| rs55723730 | 32290694 | - | A | 0.3718 | -10820 | 1 | 0.9958 | G=-,A=A | NA | | | | no | | | | no |
| rs55695092 | 32290697 | - | AA | 0.3718 | -10817 | 1 | 0.9958 | G=-,A=AA | NA | | | | no | | | | no |
| rs487649 | 32290748 | A | G | 0.3718 | -10766 | 1 | 0.9958 | G=A,A=G | NA | | | | yes | | | | no |
| **rs485774** | 32290954 | A | G | 0.3718 | -10560 | 1 | 0.9958 | G=A,A=G | NA | | | | no | | | | no |
| rs374409084 | 32291270 | AG | - | 0.3718 | -10244 | 1 | 0.9958 | G=AG,A=- | NA | | | | no | | | | no |
| rs523627 | 32291837 | G | T | 0.3718 | -9677 | 1 | 0.9958 | G=G,A=T | NA | | | | no | | | | no |
| rs525607 | 32292084 | C | T | 0.3787 | -9430 | 1 | 0.9666 | G=C,A=T | NA | | | | no | | | | no |
| rs74839053 | 32292571 | T | A | 0.3718 | -8943 | 1 | 0.9958 | G=T,A=A | NA | | | | no | | | | no |
| rs552339 | 32292715 | G | A | 0.3718 | -8799 | 1 | 0.9958 | G=G,A=A | NA | | | | no | | | | no |
| rs533885 | 32293152 | A | G | 0.3718 | -8362 | 1 | 0.9958 | G=A,A=G | NA | | | | yes | | | | no |
| rs510181 | 32293434 | A | C | 0.3787 | -8080 | 1 | 0.9666 | G=A,A=C | NA | | | | no | | | | no |
| rs2395143 | 32293578 | C | G | 0.334 | -7936 | 0.9905 | 0.835 | G=C,A=G | NA | | | | yes | | | | no |
| rs471081 | 32293598 | A | T | 0.3787 | -7916 | 0.9957 | 0.9583 | G=A,A=T | NA | | | | no | | | | no |
| rs471964 | 32293671 | G | A | 0.3638 | -7843 | 0.9913 | 0.9537 | G=G,A=A | NA | | | | no | | | | no |
| rs504703 | 32294017 | T | C | 0.3718 | -7497 | 1 | 0.9958 | G=T,A=C | NA | | | | no | | | | no |
| rs476584 | 32294192 | G | A | 0.3797 | -7322 | 1 | 0.9626 | G=G,A=A | NA | | | | yes | | | | no |
| rs501545 | 32294566 | G | A | 0.3728 | -6948 | 1 | 0.9915 | G=G,A=A | NA | | | | yes | | | | no |
| rs476885 | 32294712 | A | G | 0.3718 | -6802 | 1 | 0.9958 | G=A,A=G | NA | | | | no | | | | no |
| rs504203 | 32294843 | T | C | 0.3718 | -6671 | 1 | 0.9958 | G=T,A=C | NA | | | | yes | | | | no |
| rs505274 | 32294992 | G | A | 0.3718 | -6522 | 1 | 0.9958 | G=G,A=A | NA | | | | no | chr6:32294941-32295190 | 11 | 196 | no |
| rs508805 | 32295350 | G | A | 0.3708 | -6164 | 0.9957 | 0.9915 | G=G,A=A | NA | | | | no | | | | no |
| rs524578 | 32295357 | G | A | 0.3777 | -6157 | 0.9957 | 0.9624 | G=G,A=A | NA | | | | no | | | | no |
| rs4424116 | 32295553 | G | C | 0.3718 | -5961 | 1 | 0.9958 | G=G,A=C | NA | | | | yes | | | | no |
| rs28381583 | 32295575 | - | T | 0.4105 | -5939 | 0.9727 | 0.8005 | G=-,A=T | NA | | | | no | | | | no |
| rs6913605 | 32295694 | A | G | 0.3658 | -5820 | 1 | 0.9789 | G=A,A=G | NA | | | | yes | | | | no |
| rs77200712 | 32295747 | T | A | 0.3688 | -5767 | 0.9957 | 0.983 | G=T,A=A | NA | | | | no | | | | no |
| rs6457542 | 32295828 | A | G | 0.3708 | -5686 | 0.9957 | 0.9915 | G=A,A=G | NA | | | | yes | | | | no |
| rs6457543 | 32296029 | T | G | 0.3648 | -5485 | 1 | 0.9747 | G=T,A=G | NA | | | | no | | | | no |
| rs6457544 | 32296057 | C | T | 0.3648 | -5457 | 1 | 0.9747 | G=C,A=T | NA | | | | yes | | | | no |
| rs12528615 | 32296316 | C | T | 0.3718 | -5198 | 1 | 0.9958 | G=C,A=T | NA | | | | no | | | | no |
| rs10947252 | 32296375 | G | A | 0.3708 | -5139 | 1 | 1 | G=G,A=A | NA | | | | no | | | | no |
| rs10947253 | 32296555 | G | C | 0.3708 | -4959 | 1 | 1 | G=G,A=C | NA | | | | yes | | | | no |
| rs9348880 | 32296780 | C | T | 0.3777 | -4734 | 1 | 0.9707 | G=C,A=T | NA | | | | no | | | | no |
| rs9348881 | 32296842 | C | G | 0.3787 | -4672 | 1 | 0.9666 | G=C,A=G | NA | | | | yes | | | | no |
| rs9394086 | 32297124 | A | T | 0.3708 | -4390 | 1 | 1 | G=A,A=T | NA | | | | no | chr6:32297041-32297210 | 6 | 170 | no |
| rs9368711 | 32297156 | T | C | 0.3708 | -4358 | 1 | 1 | G=T,A=C | NA | | | | no | | | | no |
| rs9368712 | 32297328 | G | A | 0.3698 | -4186 | 1 | 0.9957 | G=G,A=A | NA | | | | yes | | | | no |
| **rs9368713** | 32297337 | T | C | 0.3708 | -4177 | 1 | 1 | G=T,A=C | NA | | | | yes | | | | no |
| rs9368714 | 32297341 | G | A | 0.326 | -4173 | 1 | 0.821 | G=G,A=A | NA | | | | no | | | | no |
| rs9380289 | 32297417 | G | A | 0.3708 | -4097 | 1 | 1 | G=G,A=A | NA | | | | yes | | | | no |
| rs146253974 | 32297641 | ATTTTAAC | - | 0.3708 | -3873 | 1 | 1 | G=AGATTTTAAC | NA | | | | no | | | | no |
| rs75052715 | 32297660 | C | G | 0.3708 | -3854 | 1 | 1 | G=C,A=G | NA | | | | no | | | | no |
| rs9296021 | 32297690 | C | T | 0.3708 | -3824 | 1 | 1 | G=C,A=T | NA | | | | no | | | | no |
| rs372016821 | 32297798 | TCTT | - | 0.3708 | -3716 | 1 | 1 | G=TCTT,A=- | NA | | | | no | | | | no |
| rs9296022 | 32297955 | G | A | 0.3708 | -3559 | 1 | 1 | G=G,A=A | NA | | | | no | | | | no |
| **rs9405090** | 32298372 | A | G | 0.3708 | -3142 | 1 | 1 | G=A,A=G | missense | p.Ser170Pro | benign | tolerated | no | | | | no |
| rs9296023 | 32298648 | A | G | 0.3688 | -2866 | 1 | 0.9915 | G=A,A=G | NA | | | | yes | | | | no |
| rs28381584 | 32298979 | - | CC | 0.3708 | -2535 | 1 | 1 | G=-,A=CC | NA | | | | no | | | | no |
| rs742582 | 32299715 | G | A | 0.3708 | -1799 | 1 | 1 | G=G,A=A | NA | | | | yes | | | | no |
| rs910052 | 32299873 | G | T | 0.3708 | -1641 | 1 | 1 | G=G,A=T | NA | | | | no | | | | no |
| rs1076711 | 32300374 | T | G | 0.3708 | -1140 | 1 | 1 | G=T,A=G | NA | | | | no | | | | no |
| rs761190 | 32300470 | T | G | 0.3708 | -1044 | 1 | 1 | G=T,A=G | NA | | | | yes | | | | no |
| rs9394087 | 32300848 | T | C | 0.3777 | -666 | 1 | 0.9707 | G=T,A=C | NA | | | | yes | | | | no |
| rs9366793 | 32301289 | T | C | 0.3787 | -225 | 1 | 0.9666 | G=T,A=C | NA | | | | no | | | | no |
| rs9348882 | 32301322 | C | T | 0.3708 | -192 | 1 | 1 | G=C,A=T | NA | | | | no | | | | no |

| | | | | | | | | | | | | | | | | | |
|---|---|---|---|---|---|---|---|---|---|---|---|---|---|---|---|---|---|
| rs36044579 | 32301369 | AC | - | 0.3708 | -145 | 1 | 1 | G=AC,A=- | NA | | | | no | | | | no |
| rs9380290 | 32301452 | C | G | 0.3777 | -62 | 1 | 0.9707 | G=C,A=G | NA | | | | yes | | | | no |
| **rs9357140** | 32301514 | G | A | 0.3708 | 0 | 1 | 1 | G=G,A=A | NA | | | | yes | chr6:32301426-32301575 | 1 | 147 | no |
| rs9391777 | 32301804 | G | C | 0.3708 | 290 | 1 | 1 | G=G,A=C | NA | | | | no | | | | no |
| rs1474729 | 32302045 | A | C | 0.3708 | 531 | 1 | 1 | G=A,A=C | NA | | | | no | | | | no |
| rs1474728 | 32302067 | C | T | 0.3708 | 553 | 1 | 1 | G=C,A=T | NA | | | | no | | | | no |
| rs9405091 | 32302539 | A | G | 0.3708 | 1025 | 1 | 1 | G=A,A=G | NA | | | | yes | | | | no |
| rs9378257 | 32302541 | T | G | 0.3708 | 1027 | 1 | 1 | G=T,A=G | NA | | | | no | | | | no |
| rs9405092 | 32302728 | T | G | 0.3708 | 1214 | 1 | 1 | G=T,A=G | NA | | | | yes | | | | no |
| rs9405093 | 32302741 | G | T | 0.3708 | 1227 | 1 | 1 | G=G,A=T | NA | | | | no | | | | no |
| rs9378258 | 32302757 | G | A | 0.3708 | 1243 | 1 | 1 | G=G,A=A | NA | | | | no | | | | no |
| rs10807100 | 32303068 | G | A | 0.3708 | 1554 | 1 | 1 | G=G,A=A | NA | | | | no | | | | no |
| rs6929776 | 32303511 | G | A | 0.3777 | 1997 | 1 | 0.9707 | G=G,A=A | NA | | | | no | | | | no |
| rs6930681 | 32304031 | G | A | 0.3708 | 2517 | 1 | 1 | G=G,A=A | NA | | | | no | | | | no |
| rs9366794 | 32304222 | C | G | 0.3708 | 2708 | 1 | 1 | G=C,A=G | NA | | | | yes | | | | no |
| rs70993817 | 32304750 | G | - | 0.3708 | 3236 | 1 | 1 | G=G,A=- | NA | | | | no | | | | no |
| rs12194915 | 32305029 | T | A | 0.3738 | 3515 | 1 | 0.9873 | G=T,A=A | NA | | | | no | | | | no |
| rs973037 | 32305125 | C | T | 0.3757 | 3611 | 1 | 0.979 | G=C,A=T | NA | | | | yes | chr6:32305061-32305210 | 1 | 117 | no |
| rs2022535 | 32305142 | C | T | 0.3777 | 3628 | 1 | 0.9707 | G=C,A=T | NA | | | | no | | | | no |
| rs973036 | 32305143 | C | G | 0.3777 | 3629 | 1 | 0.9707 | G=C,A=G | NA | | | | yes | | | | no |
| rs926594 | 32305370 | T | G | 0.3738 | 3856 | 1 | 0.9873 | G=T,A=G | NA | | | | no | | | | no |
| rs926591 | 32305690 | C | A | 0.3738 | 4176 | 1 | 0.9873 | G=C,A=A | NA | | | | no | | | | no |
| rs9368715 | 32306022 | G | A | 0.3738 | 4508 | 1 | 0.9873 | G=G,A=A | NA | | | | no | | | | no |
| rs3817982 | 32306262 | T | C | 0.3738 | 4748 | 1 | 0.9873 | G=T,A=C | NA | | | | no | | | | no |
| rs2022534 | 32307137 | T | C | 0.3738 | 5623 | 1 | 0.9873 | G=T,A=C | NA | | | | no | | | | no |
| rs2022533 | 32307260 | T | G | 0.3807 | 5746 | 1 | 0.9585 | G=T,A=G | NA | | | | no | | | | no |
| **rs1033500** | 32307382 | G | A | 0.3748 | 5868 | 1 | 0.9831 | G=G,A=A | missense | p.Pro112Leu | possibly damaging | tolerated | no | | | | no |
| rs1033498 | 32307869 | T | C | 0.3738 | 6355 | 1 | 0.9873 | G=T,A=C | NA | | | | yes | | | | no |
| rs1033497 | 32308062 | A | G | 0.3738 | 6548 | 1 | 0.9873 | G=A,A=G | NA | | | | no | | | | no |
| rs2395144 | 32308345 | T | G | 0.3738 | 6831 | 1 | 0.9873 | G=T,A=G | NA | | | | no | | | | no |
| rs4469285 | 32308642 | G | A | 0.3738 | 7128 | 1 | 0.9873 | G=G,A=A | NA | | | | yes | | | | no |
| rs7453102 | 32308690 | A | G | 0.3738 | 7176 | 1 | 0.9873 | G=A,A=G | NA | | | | yes | | | | no |
| rs2179579 | 32308734 | A | G | 0.3738 | 7220 | 1 | 0.9873 | G=A,A=G | NA | | | | yes | | | | no |
| rs2179578 | 32308770 | G | A | 0.3738 | 7256 | 1 | 0.9873 | G=G,A=A | NA | | | | yes | | | | no |
| rs2143468 | 32309003 | A | T | 0.3767 | 7489 | 1 | 0.9748 | G=A,A=T | NA | | | | no | | | | no |
| rs2143467 | 32309296 | C | G | 0.3738 | 7782 | 1 | 0.9873 | G=C,A=G | NA | | | | yes | | | | no |
| **rs2143466** | 32309323 | C | T | 0.3867 | 7809 | 1 | 0.9346 | G=C,A=T | NA | | | | yes | | | | no |
| rs2143465 | 32309352 | T | C | 0.3738 | 7838 | 1 | 0.9873 | G=T,A=C | NA | | | | yes | | | | no |
| rs2143464 | 32309434 | T | C | 0.3738 | 7920 | 1 | 0.9873 | G=T,A=C | NA | | | | yes | | | | no |
| rs9268256 | 32309864 | C | T | 0.3738 | 8350 | 1 | 0.9873 | G=C,A=T | NA | | | | no | | | | no |
| rs9268257 | 32310103 | G | A | 0.3738 | 8589 | 1 | 0.9873 | G=G,A=A | NA | | | | yes | | | | no |
| rs9268258 | 32310684 | G | A | 0.3738 | 9170 | 1 | 0.9873 | G=G,A=A | NA | | | | no | chr6:32310681-32310830 | 1 | 101 | no |
| rs9268259 | 32310803 | G | A | 0.3738 | 9289 | 1 | 0.9873 | G=G,A=A | NA | | | | no | chr6:32310681-32310830 | 1 | 101 | no |
| rs9268260 | 32310967 | C | T | 0.3738 | 9453 | 1 | 0.9873 | G=C,A=T | NA | | | | yes | | | | no |
| rs9268261 | 32310986 | C | G | 0.3738 | 9472 | 1 | 0.9873 | G=C,A=G | NA | | | | yes | | | | no |
| rs1555117 | 32311148 | C | G | 0.3738 | 9634 | 1 | 0.9873 | G=C,A=G | NA | | | | yes | | | | no |
| rs2395145 | 32311871 | T | A | 0.3738 | 10357 | 1 | 0.9873 | G=T,A=A | NA | | | | no | | | | no |
| rs2395146 | 32311875 | T | A | 0.3738 | 10361 | 1 | 0.9873 | G=T,A=A | NA | | | | no | | | | no |
| rs1555116 | 32312740 | C | A | 0.3738 | 11226 | 1 | 0.9873 | G=C,A=A | NA | | | | no | | | | no |
| rs4576282 | 32312796 | A | G | 0.3738 | 11282 | 1 | 0.9873 | G=A,A=G | NA | | | | no | | | | no |
| rs9268271 | 32312883 | C | T | 0.3738 | 11369 | 1 | 0.9873 | G=C,A=T | NA | | | | yes | | | | no |
| rs4959024 | 32313010 | C | T | 0.3738 | 11496 | 1 | 0.9873 | G=C,A=T | NA | | | | no | | | | no |
| rs4959092 | 32313011 | T | G | 0.3738 | 11497 | 1 | 0.9873 | G=T,A=G | NA | | | | yes | | | | no |
| rs4959093 | 32313097 | T | C | 0.3738 | 11583 | 1 | 0.9873 | G=T,A=C | NA | | | | yes | | | | no |
| rs4959094 | 32313136 | C | T | 0.3738 | 11622 | 1 | 0.9873 | G=C,A=T | NA | | | | no | | | | no |
| rs4959025 | 32313143 | A | C | 0.3738 | 11629 | 1 | 0.9873 | G=A,A=C | NA | | | | yes | | | | no |
| rs4959026 | 32313461 | G | T | 0.3738 | 11947 | 1 | 0.9873 | G=G,A=T | NA | | | | no | | | | no |
| rs4959096 | 32314676 | G | A | 0.3738 | 13162 | 1 | 0.9873 | G=G,A=A | NA | | | | no | | | | no |
| rs9268283 | 32315173 | T | C | 0.3738 | 13659 | 1 | 0.9873 | G=T,A=C | NA | | | | yes | | | | no |
| rs9268284 | 32315216 | G | A | 0.3738 | 13702 | 1 | 0.9873 | G=G,A=A | NA | | | | no | | | | no |
| rs9268285 | 32315557 | T | C | 0.3738 | 14043 | 1 | 0.9873 | G=T,A=C | NA | | | | yes | | | | no |
| rs9268286 | 32315593 | C | G | 0.3738 | 14079 | 1 | 0.9873 | G=C,A=G | NA | | | | no | | | | no |
| rs2076542 | 32317127 | G | A | 0.3738 | 15613 | 1 | 0.9873 | G=G,A=A | NA | | | | no | | | | no |
| rs2076541 | 32317215 | T | C | 0.3738 | 15701 | 1 | 0.9873 | G=T,A=C | NA | | | | no | | | | no |
| rs2076540 | 32317276 | T | C | 0.3678 | 15762 | 0.9871 | 0.962 | G=T,A=C | NA | | | | no | | | | no |
| rs2076538 | 32317471 | A | C | 0.3738 | 15957 | 1 | 0.9873 | G=A,A=C | NA | | | | no | | | | no |
| rs143741085 | 32317922 | TTTC | - | 0.3738 | 16408 | 1 | 0.9873 | G=TTTC,A=- | NA | | | | no | | | | no |
| rs761187 | 32318036 | A | G | 0.3678 | 16522 | 0.9871 | 0.962 | G=A,A=G | NA | | | | no | | | | no |
| rs2143463 | 32318729 | A | G | 0.3738 | 17215 | 1 | 0.9873 | G=A,A=G | NA | | | | yes | | | | no |
| rs9268301 | 32319637 | G | A | 0.327 | 18123 | 1 | 0.8247 | G=G,A=A | NA | | | | yes | | | | no |
| rs9268307 | 32326531 | T | C | 0.3738 | 25017 | 1 | 0.9873 | G=T,A=C | NA | | | | no | | | | no |
| rs9268326 | 32330153 | T | C | 0.3738 | 28639 | 1 | 0.9873 | G=T,A=C | NA | | | | yes | | | | no |
| rs9268327 | 32330164 | C | G | 0.3767 | 28650 | 1 | 0.9748 | G=C,A=G | NA | | | | yes | | | | no |
| rs9268329 | 32330174 | A | G | 0.3777 | 28660 | 1 | 0.9707 | G=A,A=G | NA | | | | no | | | | no |
| rs9268333 | 32330651 | C | T | 0.3738 | 29137 | 1 | 0.9873 | G=C,A=T | NA | | | | no | | | | no |
| rs6934200 | 32331069 | T | C | 0.3738 | 29555 | 1 | 0.9873 | G=T,A=C | NA | | | | no | | | | no |
| rs6912095 | 32331429 | C | A | 0.3738 | 29915 | 1 | 0.9873 | G=C,A=A | NA | | | | no | | | | no |
| rs9268343 | 32331824 | A | G | 0.3728 | 30310 | 0.9957 | 0.9831 | G=A,A=G | NA | | | | no | | | | no |
| rs9268344 | 32331837 | C | T | 0.3728 | 30323 | 0.9957 | 0.9831 | G=C,A=T | NA | | | | no | | | | no |
| rs9268345 | 32331841 | G | A | 0.3728 | 30327 | 0.9957 | 0.9831 | G=G,A=A | NA | | | | no | | | | no |
| rs9268346 | 32331927 | C | G | 0.3738 | 30413 | 1 | 0.9873 | G=C,A=G | NA | | | | no | | | | no |
| rs9268357 | 32332996 | C | G | 0.3738 | 31482 | 1 | 0.9873 | G=C,A=G | NA | | | | no | | | | no |
| rs9268363 | 32333410 | A | C | 0.3738 | 31896 | 1 | 0.9873 | G=A,A=C | NA | | | | no | | | | no |
| **rs9268368** | 32333955 | T | C | 0.3748 | 32441 | 1 | 0.9831 | G=T,A=C | missense | p.Tyr69Cys | benign | deleterious | no | | | | no |
| rs9268369 | 32334224 | C | G | 0.3738 | 32710 | 1 | 0.9873 | G=C,A=G | NA | | | | no | | | | no |
| rs9268370 | 32334258 | G | T | 0.3738 | 32744 | 1 | 0.9873 | G=G,A=T | NA | | | | yes | | | | no |
| rs9268373 | 32334489 | G | A | 0.3738 | 32975 | 1 | 0.9873 | G=G,A=A | NA | | | | yes | | | | no |
| rs2073046 | 32336074 | C | T | 0.3738 | 34560 | 1 | 0.9873 | G=C,A=T | NA | | | | no | | | | no |
| **rs9268384** | 32336586 | A | G | 0.3738 | 35072 | 1 | 0.9873 | G=A,A=G | missense | p.Tyr36His | benign | deleterious | yes | | | | no |
| rs9268386 | 32337234 | G | A | 0.3738 | 35720 | 1 | 0.9873 | G=G,A=A | NA | | | | no | | | | no |
| rs9268387 | 32337367 | T | G | 0.3738 | 35853 | 1 | 0.9873 | G=T,A=G | NA | | | | no | | | | no |
| rs9268394 | 32338386 | G | C | 0.3738 | 36872 | 1 | 0.9873 | G=G,A=C | NA | | | | no | | | | no |

**Supplementary Table 11.** The SNPs located within or close to the *C6orf10/LOC101929163* locus (chr6: 32213638–32338386) reported to be associated with neurodegenerative or autoimmune diseases.

| SNP | $R^2$ associated with rs9357140 | Candidate gene | p-value | Reported odds ratio (95% CI)* | Disease | Reference |
|---|---|---|---|---|---|---|
| rs9268877 | 0 | *HLA-DRA/HLA-DRB5* | $1\times10^{-8}$ | 1.204(1.11-1.3) | FTD | PMID:24943344 |
| rs9268856 | 0.06 | *HLA-DRA/HLA-DRB5* | $5.51\times10^{-9}$ | 0.8(0.76-0.86) | FTD | PMID:24943344 |
| rs9271192 | 0.07 | *HLA-DRB1* | 0.004 | 2.069(1.26-3.43) | AD | PMID:29190991 |
| rs3135388 | 0.06 | *HLA-DRA* | $8.94\times10^{-81}$ | 1.99(1.84-2.15) | Multiple Sclerosis | PMID: 17660530 |
| rs3129871 | 0.27 | *HLA-DRA* | $5.7\times10^{-15}$ | 1.72(1.59-1.86) | Multiple Sclerosis | PMID: 23472185 |
| rs3129882 | 0.03 | *HLA-DRA* | $1.9\times10^{-10}$ | 1.26(1.17-1.35) | Parkinson's disease | PMID: 20711177 |
| rs3129934 | 0.1 | *C6orf10* | $9\times10^{-11}$ | 3.3(2.3-4.9) | Multiple Sclerosis | PMID: 22457343 |
| rs2395148 | 0.03 | *C6orf10* | $2\times10^{-10}$ | 5.37(3.02-9.56) | Arthritis | PMID: 18576341 |
| rs6910071 | 0.39 | *C6orf10* | $1\times10^{-299}$ | 2.88(2.73-3.03) | Rheumatoid arthritis | PMID: 20453842 |
| rs9296015 | 0.16 | *NOTCH4* | $1\times10^{-8}$ | 1.85(1.49-2.27) | Systemic sclerosis | PMID: 21779181 |
| rs443198 | 0.008 | *NOTCH4* | $9\times10^{-21}$ | 1.82(1.59-2.04) | Systemic sclerosis | PMID:21779181 |
| rs2273017 | 0.45 | *C6orf10* | $2\times10^{-22}$ | 1.53(1.40-1.66) | Grave's disease | PMID: 21900946 |
| rs404860 | 0.0005 | *NOTCH4* | $4\times10^{-23}$ | 1.21(1.16-1.25) | Asthma | PMID: 21804548 |

*SNPs with an OR ≥1.2 or ≤ 0.8 were included.

**Supplementary Table 12.** The distribution of phenotypes for *C9orf72* carriers in the discovery and replication stages.

| | ALS | FTD | FTD-ALS | Asymptomatic | Total number |
|---|---|---|---|---|---|
| **Discovery stage** | 61 (42.4%) | 40 (27.8%) | 21 (14.5%) | 22 (15.3%) | 144 |
| **Replication stage** | 0 | 136 (72.7%) | 51 (27.3%) | 0 | 187 |

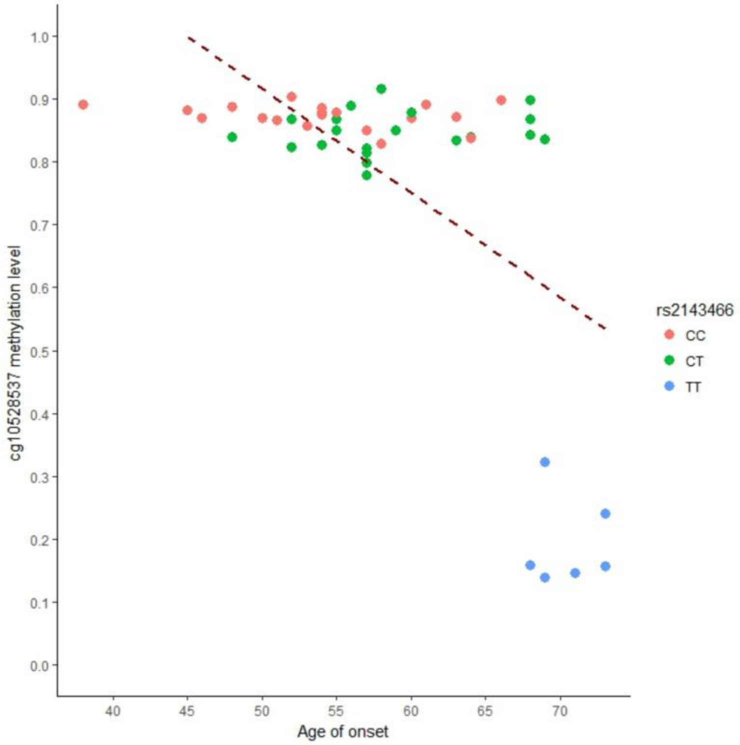


**Supplementary Figure 1.** Genotypes of rs2143466 in the discovery cohort are significantly associated with DNA methylation status ($p<1.0\times10^{-6}$, B=-0.25) and AO ($p=7.0\times10^{-6}$ adjusted for sex and *TMEM106B* genotypes, B=7.1).

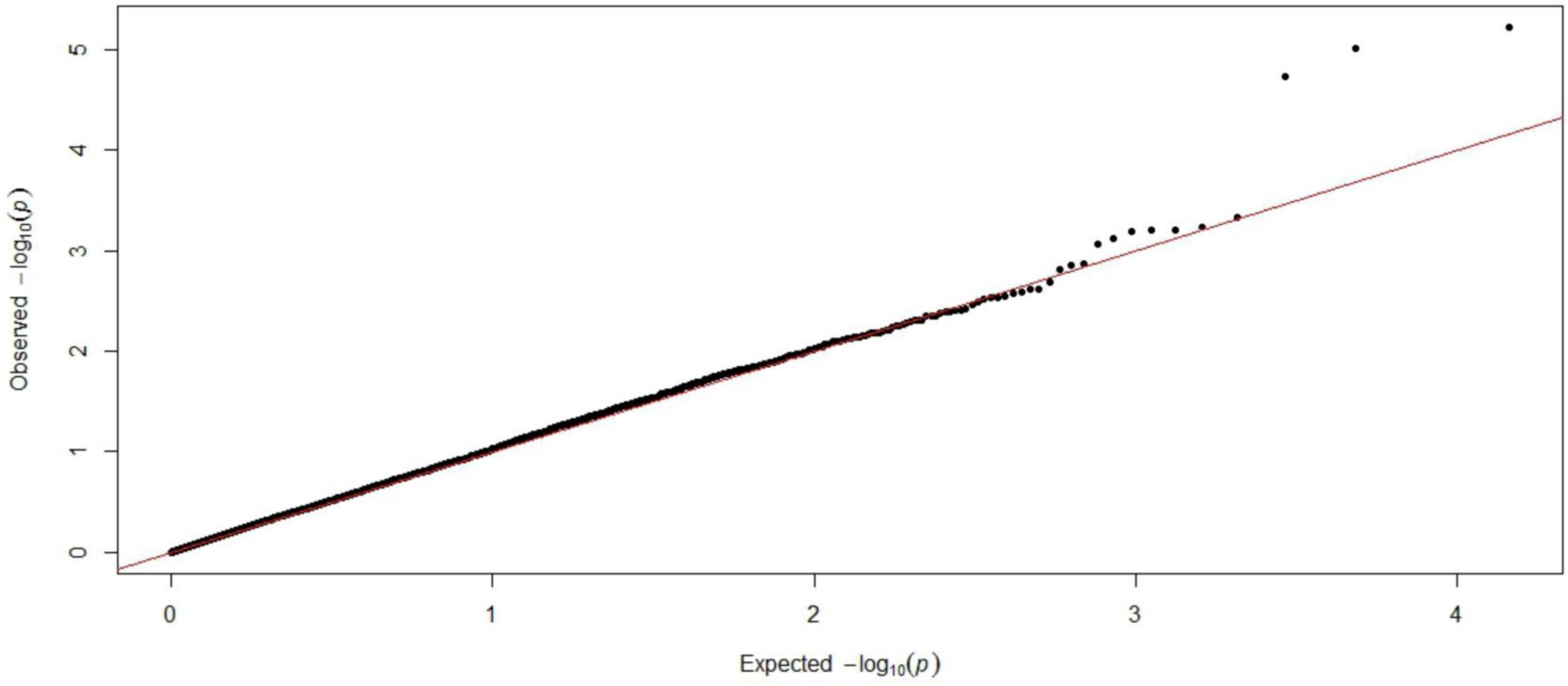


**Supplementary Figure 2.** A Q-Q plot of the genome-wide DNA methylation analysis of CpG-SNPs.

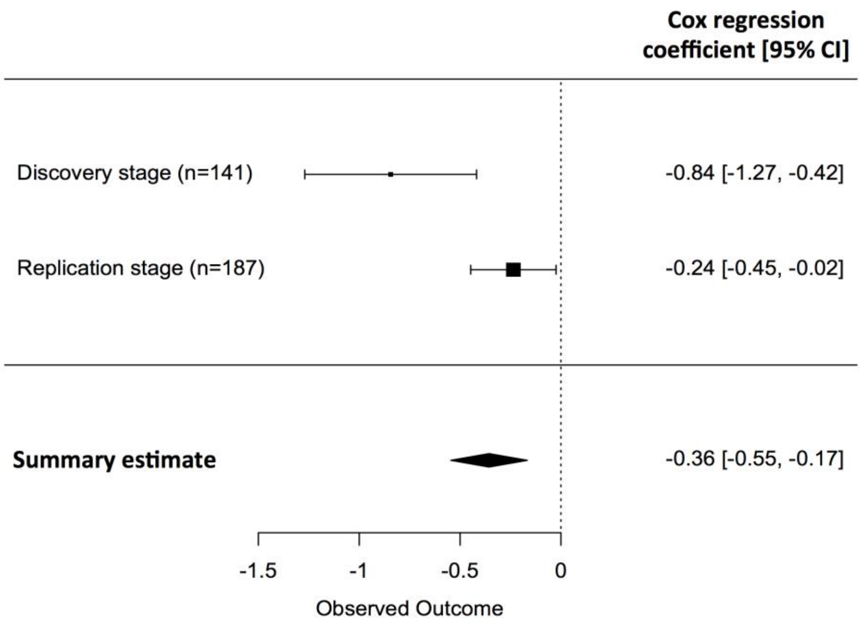


**Supplementary Figure 3.** The result of the Cox regression coefficient meta-analysis of rs2143466 for the discovery (n=141) and replication (n=187) cohorts; the regression coefficient equals logHR; CI = confidence interval.

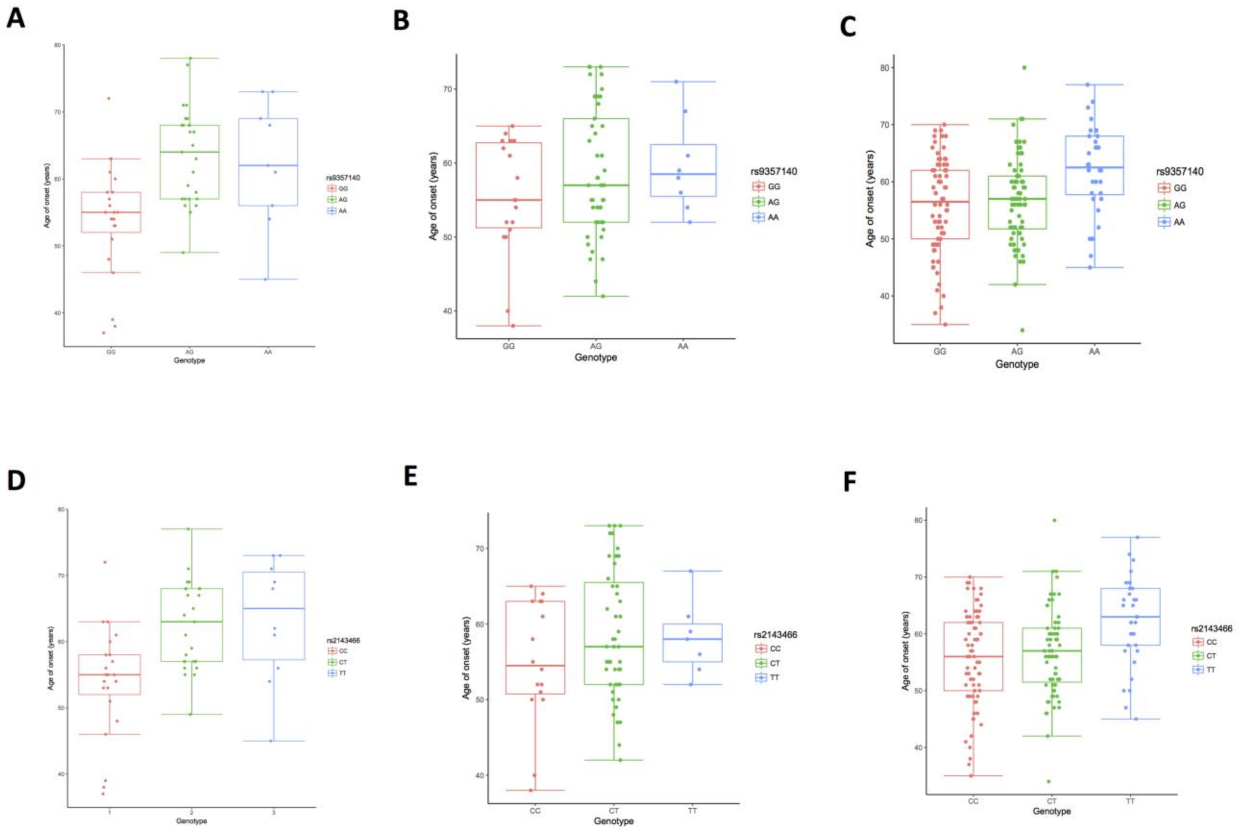


**Supplementary Figure 4.** (**A-C**) Box-Scatter plots representing the association between rs9357140 genotypes and age of onset in *C9orf72* patients with ALS (**A**): adjusted p=0.002, B=4.0 (SE: 1.53); FTD-ALS (**B**): adjusted p=0.125, B=2.63(SE:1.69); and FTD (**C**): adjusted p=0.0008, B=2.82 (SE: 0.83). (**D-F**) Box-Scatter plots representing the association between rs2143466 genotypes and age of onset in *C9orf72* patients with ALS (**D**): adjusted p=0.0007, B=5.1 (SE: 1.43); FTD-ALS (**E**): adjusted p=0.199, B=2.3 (SE:1.77); and FTD (**F**): adjusted p=0.0004, B=2.95 (SE: 0.82).

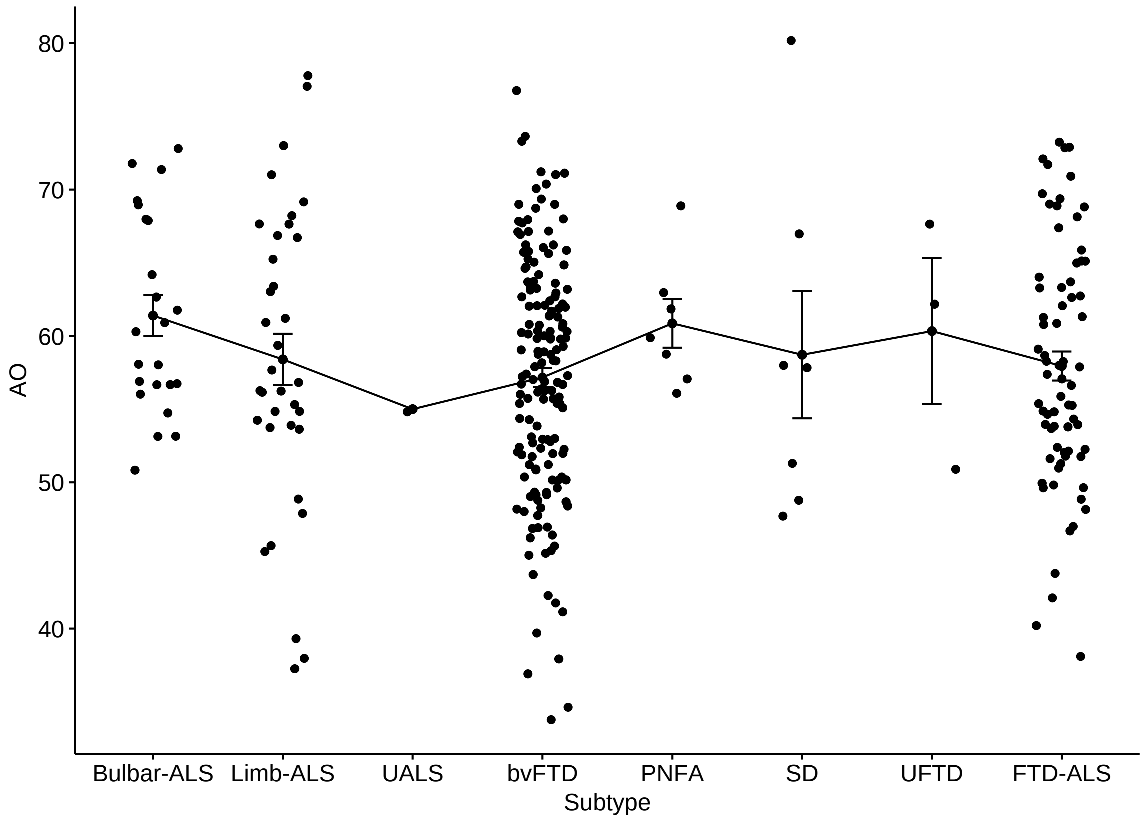


**Supplementary Figure 5**. Scatter plot of age of onset (AO) in disease subtypes of unrelated *C9orf72* patients (total n=304). Mean AO and standard error bar for each group are presented. We found no significant difference in AO among the ALS subtypes (bulbar ALS, limb ALS, unspecified ALS) or FTD subtypes (bvFTD, SD, PNFA, unspecified FTD): p>0.05, Kruskal Wallis test.

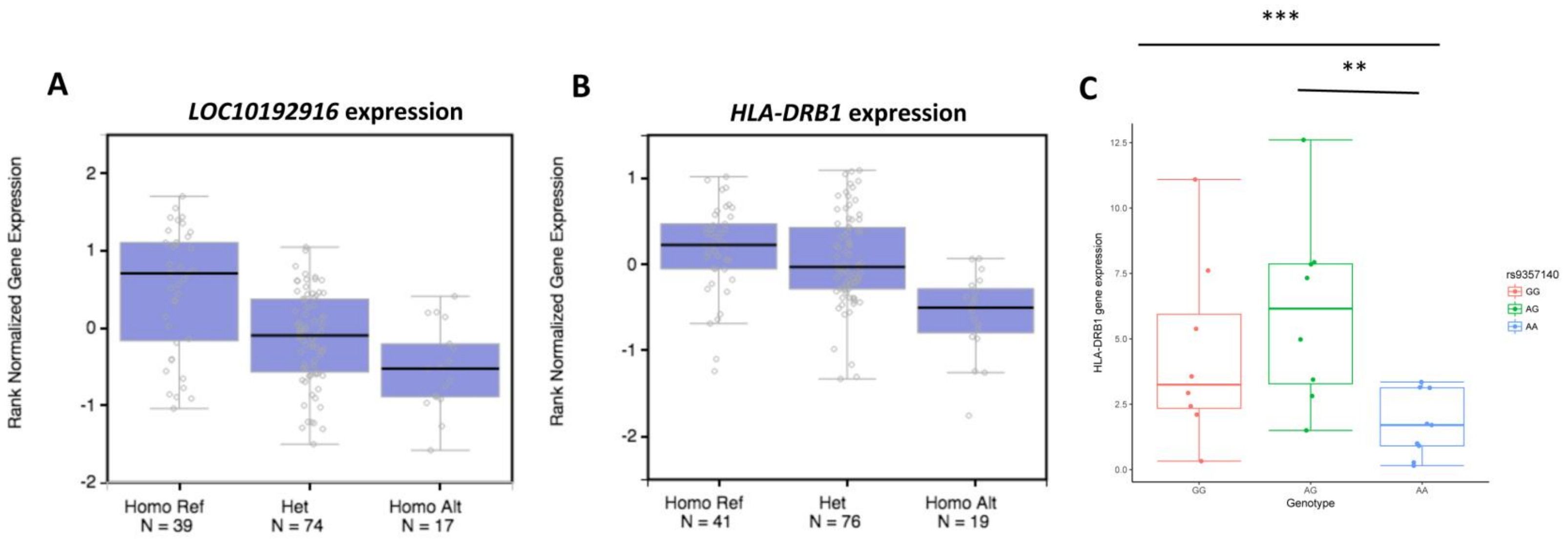


**Supplementary Figure 6.** Results of the association study of rs9357140 using GTEx eQTL data (Ref=G-allele, Alt=A-allele) and quantitative RT-PCR. Genotypes of rs9357140 are significantly associated with (**A**) *LOC101929163* expression in nucleus accumbens ($p=7.6\times10^{-6}$, n=130) and (**B)** *HLA-DRB1* expression in frontal cortex tissues ($p=4.1\times10^{-6}$, n=136). (**C**) In 25 ALS frontal cortex tissues, rs9357140 AA-carriers have reduced *HLA-DRB1* expression compared to AG- or GG-carriers ($^{**}$p=0.001 for AA vs AG; $^{***}$p=0.000003 for AA vs GG; Mann-Whitney U test).

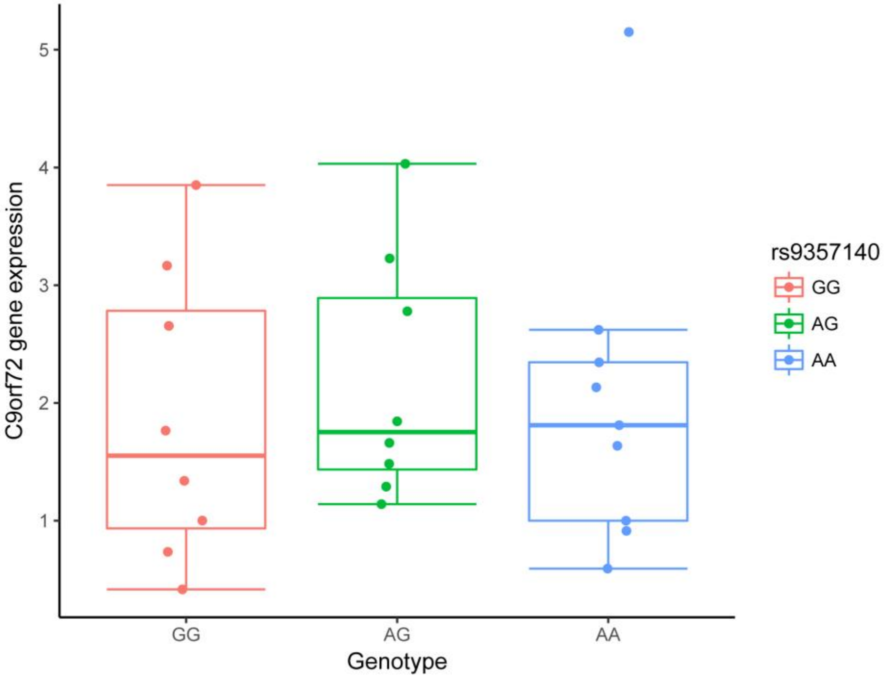


**Supplementary Figure 7.** Genotypes of rs9357140 are not associated with *C9orf72* expression (p>0.05, Mann-Whitney U test) in frontal cortex tissues from 25 ALS cases. Expression *C9orf72* and *HLA-DRB1* are not significantly correlated with each other (p=0.23, multivariate linear regression, adjusted for sex).

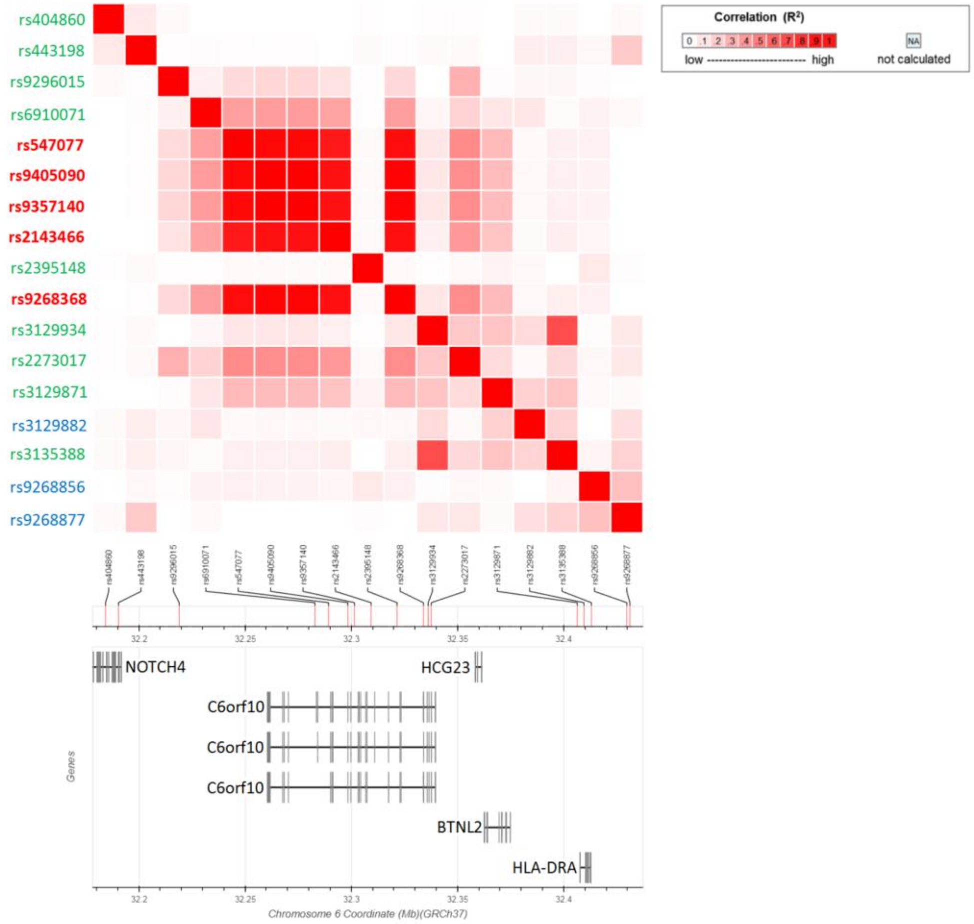


**Supplementary Figure 8.** LD-plot with the location of the genetic variations (rs547077, rs9405090, rs9357140, rs2143466, and rs9268368) significantly associated with age of onset in *C9orf72* patients (in red font), as well as nearby top-significant variants (rs404860, rs443198, rs9296015, rs6910071, rs2395148, rs3129934, rs2273017, rs3129871, rs3129882, rs3135388, rs9268856, and rs9268877) reported to be linked to neurodegenerative (blue font) or autoimmune (green font) diseases (Supplementary Table 11).

**APPENDIX**

**Acknowledgments for the International FTD-Genomics Consortium (IFGC)**

Intramural funding from the National Institute of Neurological Disorders and Stroke (NINDS) and National Institute on Aging (NIA), the Wellcome/MRC Centre on Parkinson's disease, Alzheimer's Research UK (ARUK, Grant ARUK-PG2012-18) and by the office of the Dean of the School of Medicine, Department of Internal Medicine, at Texas Tech University Health Sciences Center.
We thank Mike Hubank and Kerra Pearce at the Genomic core facility at the Institute of Child Health (ICH), University College of London (UCL), for assisting RF in performing Illumina genotyping experiments (FTD-GWAS genotyping). This study utilized the high-performance computational capabilities of the Biowulf Linux cluster at the National Institutes of Health, Bethesda, Md. (http://biowulf.nih.gov). North American Brain Expression Consortium (NABEC) - The work performed by the North American Brain Expression Consortium (NABEC) was supported in part by the Intramural Research Program of the National Institute on Aging, National Institutes of Health, part of the US Department of Health and Human Services; project number ZIA AG000932-04. In addition this work was supported by a Research Grant from the Department of Defense, W81XWH-09-2-0128. UK Brain Expression Consortium (UKBEC) - This work performed by the UK Brain Expression Consortium (UKBEC) was supported by the MRC through the MRC Sudden Death Brain Bank (C.S.), by a Project Grant (G0901254 to J.H. and M.W.) and by a Fellowship award (G0802462 to M.R.). D.T. was supported by the King Faisal Specialist Hospital and Research Centre, Saudi Arabia. Computing facilities used at King's College London were supported by the National Institute for Health Research (NIHR) Biomedical Research Centre based at Guy's and St Thomas' NHS Foundation Trust and King's College London. We would like to thank AROS Applied Biotechnology AS company laboratories and Affymetrix for their valuable input. RF's work is supported by Alzheimer's Society (grant number 284), UK; JBJK was supported by the National Health and Medical Resarch Council (NHMRC) Australia, Project Grants 510217 and 1005769; CDS was supported by NHMRC Project Grants 630428 and 1005769; PRS was supported by NHMRC Project Grants 510217 and 1005769 and acknowledges that DNA samples were prepared by Genetic Repositories Australia, supported by NHMRC Enabling Grant 401184; GMH was supported by NHMRC Research Fellowship 630434, Project Grant 1029538, Program Grant 1037746; JRH was supported by the Australian Research Council Federation Fellowship, NHMRC Project Grant 1029538, NHMRC Program Grant 1037746; OP was supported by NHMRC Career Development Fellowship 1022684, Project Grant 1003139. IH, AR and MB acknowledge the patients and controls who participated in this project and the Trinitat Port-Carbó and her family who are supporting Fundació ACE research programs. CC was supported by Grant P30-NS069329-01 and acknowledges that the recruitment and clinical characterization of research participants at Washington University were supported by NIH P50 AG05681, P01 AG03991, and P01 AG026276. LB and GB were supported by the Ricerca Corrente, Italian Ministry of Health; RG was supported by Fondazione CARIPLO 2009-2633, Ricerca Corrente, Italian Ministry of Health; GF was supported by Fondazione CARIPLO 2009-2633. ES was supported by the Italian Ministry of Health; CF was supported by Fondazione Cariplo; MS was supported from the

Italian Ministry of Health (Ricerca Corrente); MLW was supported by Government funding of clinical research within NHS Sweden (ALF); KN was supported by Thure Carlsson Foundation; CN was supported by Swedish Alzheimer Fund. IRAM and GYRH were supported by CIHR (grant 74580) PARF (grant C06-01). JG was supported by the NINDS intramural research funds for FTD research. CMM was supported by Medical Research Council UK, Brains for Dementia Research, Alzheimer's Society, Alzheimer's Research UK, National Institutes for Health Research, Department of Health, Yvonne Mairy Bequest and acknowledges that tissue made available for this study was provided by the Newcastle Brain Tissue Resource, which was funded in part by grants G0400074 and G1100540 from the UK MRC, the Alzheimer's Research Trust and Alzheimer's Society through the Brains for Dementia Research Initiative and an NIHR Biomedical Research Centre Grant in Ageing and Health, and NIHR Biomedical Research Unit in Lewy Body Disorders. CMM was supported by the UK Department of Health and Medical Research Council and the Research was supported by the National Institute for Health Research Newcastle Biomedical Research Centre based at Newcastle Hospitals Foundation Trust and Newcastle University and acknowledges that the views expressed are those of the authors and not necessarily those of the NHS, the NIHR or the Department of Health; JA was supported by MRC, Dunhill Medical Trust, Alzheimer's Research UK; TDG was supported by Wellcome Trust Senior Clinical Fellow; IGM was supported by NIHR Biomedical Research Centre and Unit on Ageing Grants and acknowledges the National Institute for Health Research Newcastle Biomedical Research Centre based at Newcastle Hospitals Foundation Trust and Newcastle University. The views expressed are those of the author(s) and not necessarily those of the NHS, the NIHR or the Department of Health; AJT was supported by Medical Research Council, Alzheimer's Society, Alzheimer's Research UK, National Institutes for Health Research. EJ was supported by NIHR, Newcastle Biomedical Research Centre. PP, CR, SOC and EA were supported partially by FIMA (Foundation for Applied Medical Research); PP acknowledges Manuel Seijo-Martínez (Department of Neurology, Hospital do Salnés, Pontevedra, Spain), Ramon Rene, Jordi Gascon and Jaume Campdelacreu (Department of Neurology, Hospital de Bellvitge, Barcelona, Spain) for providing FTD DNA samples. RP, JDS, PA and AK were supported by German Federal Ministry of Education and Research (BMBF; grant number FKZ 01GI1007A – German FTLD consortium). IR was supported by Ministero dell'Istruzione, dell'Università e della Ricerca (MIUR) of Italy. PStGH was supported by the Canadian Institutes of Health Research, Wellcome Trust, Ontario Research Fund. FT was supported by the Italian Ministry of Health (ricerca corrente) and MIUR grant RBAP11FRE9; GR and GG were supported by the Italian Ministry of Health (ricerca corrente). JBR was supported by Camrbidge NIHR Biomedical Research Centre and Wellcome Trust (088324). JU, JC, SM were supported by the MRC Prion Unit core funding and acknowledge MRC UK, UCLH Biomedical Research Centre, Queen Square Dementia BRU; SM acknowledges the work of John Beck, Tracy Campbell, Gary Adamson, Ron Druyeh, Jessica Lowe, Mark Poulter. AD acknowledges the work of Benedikt Bader and of Manuela Neumann, Sigrun Roeber, Thomas Arzberger and Hans Kretzschmar†; VMVD and JQT were supported by Grants AG032953, AG017586 and AG010124; MG was supported by Grants AG032953, AG017586, AG010124 and NS044266; VMVD acknowledges EunRan Suh, PhD for assistance with sample handling and Elisabeth McCarty-Wood for help in selection of cases; JQT acknowledges Terry Schuck, John Robinson and Kevin Raible for assistance with neuropathological evaluation of cases. CVB and the Antwerp site were in part funded by the MetLife Foundation for Medical Research Award (to CVB); the Belgian Science Policy Office (BELSPO) Interuniversity Attraction Poles program; the Flemish Government initiated Methusalem Excellence Program (to CVB); the Flemish government initiated Impulse Program on

Networks for Dementia Research (VIND); the Research Foundation Flanders (FWO) and the University of Antwerp Research Fund. CVB and JvdZ acknowledge the neurologists S Engelborghs, PP De Deyn, A Sieben, R Vandenberghe and the neuropathologist JJ Martin for the clinical and pathological diagnoses. CVB and JvdZ further thank the personnel of the Neuromics Support Facility of the VIB Center for Molecular Neurology and the Antwerp Biobank of the Institute Born-Bunge for their expert support. IL and AB were supported by the program "Investissements d'avenir" ANR-10-IAIHU-06 and acknowledges the contribution of The French research network on FTLD/FTLD-ALS for the contribution in samples collection. BN is founded by Fondazione Cassa di Risparmio di Pistoia e Pescia (grant 2014.0365), SS is founded by the Cassa di Risparmio di Firenze (grant 2014.0310) and a grant from Ministry of Health n° RF-2010-2319722. JEN was supported by the Novo Nordisk Foundation, Denmark. MR was supported by the German National Genome Network (NGFN); German Ministry for Education and Research Grant Number 01GS0465. JDR, MNR, NCF and JDW were supported by an MRC programme grant and the Dementia Platform UK, the NIHR Queen Square Dementia Biomedical Research Unit (BRU) and the Leonard Wolfson Experimental Neurology Centre. MGS was supported by MRC grant n G0301152, Cambridge Biomedical Research Centre and acknowledges Mrs K Westmore for extracting DNA. HM was supported by the Motor Neuron Disease Association (Grant 6057). RR was supported by P50 AG016574, R01 NS080882, R01 NS065782, P50 NS72187 and the Consortium for Frontotemporal Dementia; DWD was supported by P50NS072187, P50AG016574, State of Florida Alzheimer Disease Initiative, & CurePSP, Inc.; NRGR, JEP, RCP, DK, BFB were supported by P50 AG016574; KAJ was supported by R01 AG037491; WWS was supported by NIH AG023501, AG019724, Consortium for Frontotemporal Dementia Research; BLM was supported by P50AG023501, P01AG019724, Consortium for FTD Research; HR was supported by AG032306. JCvS was supported by Stichting Dioraphte Foundation (11 02 03 00), Nuts Ohra Foundation (0801-69), Hersenstichting Nederland (BG 2010-02) and Alzheimer Nederland. CG and HHC acknowledge families, patients, clinicians including Dr Inger Nennesmo and Dr Vesna Jelic, Professor Laura Fratiglioni for control samples and Jenny Björkström, Håkan Thonberg, Charlotte Forsell, Anna-Karin Lindström and Lena Lilius for sample handling. CG was supported by Swedish Brain Power (SBP), the Strategic Research Programme in Neuroscience at Karolinska Institutet (StratNeuro), the regional agreement on medical training and clinical research (ALF) between Stockholm County Council and Karolinska Institutet, Swedish Alzheimer Foundation, Swedish Research Council, Karolinska Institutet PhD-student funding, King Gustaf V and Queen Victoria's Free Mason Foundation. FP, AR, VD and FL acknowledge Labex DISTALZ. RF acknowledges the help and support of Mrs. June Howard at the Texas Tech University Health Sciences Center Office of Sponsored Programs for tremendous help in managing Material Transfer Agreement at TTUHSC.
